# Identification and regulation of circulating tumor-TCR-matched cytotoxic CD4^+^ lymphocytes by KLRG1 in bladder cancer

**DOI:** 10.1172/jci.insight.177373

**Published:** 2025-04-29

**Authors:** Serena S. Kwek, Hai Yang, Tony Li, Arielle Ilano, Eric D. Chow, Li Zhang, Hewitt Chang, Diamond Luong, Averey Lea, Matthew Clark, Alec Starzinski, Yimin Shi, Elizabeth McCarthy, Sima Porten, Maxwell V. Meng, Chun Jimmie Ye, Lawrence Fong, David Y. Oh

**Affiliations:** 1 Division of Hematology/Oncology, Department of Medicine,; 2 Helen Diller Family Comprehensive Cancer Center Biostatistics and Population Research Core,; 3 Center for Advanced Technology,; 4 Department of Epidemiology and Biostatistics,; 5 Helen Diller Family Comprehensive Cancer Center,; 6 Division of Rheumatology,; 7 Department of Urology, Department of Medicine, Department of Epidemiology and Biostatistics, and Institute for Human Genetics,; 8 Parker Institute for Cancer Immunotherapy, and; 9 Bakar Computational Health Sciences Institute, UCSF, San Francisco, California, USA.; 10 Chan Zuckerberg Biohub, San Francisco, California, USA.; 11 Fred Hutchinson Cancer Center, Seattle, Washington, USA.

**Keywords:** Clinical trials, Immunology, Oncology, Cancer immunotherapy, Cellular immune response, T cells

## Abstract

While cytotoxic CD4^+^ tumor-infiltrating lymphocytes have anticancer activity in patients, whether these can be noninvasively monitored and how these are regulated remains obscure. By matching single cells with T cell receptors (TCRs) in tumor and blood of patients with bladder cancer, we identified distinct pools of tumor-matching cytotoxic CD4^+^ T cells in the periphery directly reflecting the predominant antigenic specificities of intratumoral CD4^+^ tumor-infiltrating lymphocytes. On one hand, the granzyme B–expressing (GZMB-expressing) cytotoxic CD4^+^ subset proliferated in blood in response to PD-1 blockade but was separately regulated by the killer cell lectin-like receptor G1 (KLRG1), which inhibited their killing by interacting with E-cadherin. Conversely, a clonally related, GZMK-expressing circulating CD4^+^ population demonstrated basal proliferation and a memory phenotype that may result from activation of GZMB^+^ cells, but was not directly mobilized by PD-1 blockade. As KLRG1 marked the majority of circulating tumor-TCR-matched cytotoxic CD4^+^ T cells, this work nominates KLRG1 as a means to isolate them from blood and provide a window into intratumoral CD4^+^ recognition, as well as a putative regulatory receptor to mobilize the cytolytic GZMB^+^ subset for therapeutic benefit. Our findings also underscore ontogenic relationships of GZMB- and GZMK-expressing populations and the distinct cues that regulate their activity.

## Abstract 2

Cytotoxic CD4^+^ circulate between the blood and tumor of bladder cancer patients, where they exist as clonally related but distinct pools of granzyme B+ effector cells dually inhibited by PD-1 and KLRG1, and effector memory granzyme K+ cells that are not mobilized by PD-1 blockade.

## Introduction

The presence of cytotoxic CD4^+^ T cells with T helper 1 (Th1) phenotype and function has been documented in both mice and humans (1). In cancer, cytotoxic CD4^+^ T cells have been implicated in tumor killing after CTLA-4 blockade in lymphopenic murine hosts (2–5) and in patients with melanoma after ipilimumab (6). However, the functional program that regulates the killing activity of cytotoxic CD4^+^ T cells in patients with cancer has yet to be fully elucidated. We have previously found CD4^+^ T cells that are cytotoxic and express granzymes and perforin, using single-cell RNA sequencing (scRNA-seq) and T cell receptor sequencing (scTCR-seq) of patients with muscle-invasive bladder cancer (7). We found that cytotoxic CD4^+^ T cells were enriched and clonally expanded in tumors. Their relevance for antitumor responses was demonstrated by 2 findings: First, a signature of proliferative cytotoxic CD4^+^ T cells in tumors predicted clinical response in patients with metastatic bladder cancer treated with anti–PD-L1. Second, cytotoxic CD4^+^ T cells from the tumors could kill autologous bladder tumors by recognizing major histocompatibility complex (MHC) class II molecules. Similar findings were obtained from scRNA-seq and single-cell cytotoxicity assays demonstrating MHC class II–dependent tumor killing in patients with melanoma (8). In both studies, discrimination of cytotoxic CD4^+^ T cells with tumor cytolytic activity was enabled by flow cytometric sorting of CD4 protein–expressing cells, to distinguish them from conventional CD8^+^ T cells.

While cytotoxic CD4^+^ T cells are therefore an important constituent of the tumor microenvironment, it remains unclear whether these cells can act outside of tumors, specifically whether these cytotoxic CD4^+^ T cells can circulate between tumor and blood, and what regulates their function in the periphery. This is an important question, as circulating, blood cytotoxic CD4^+^ T cells that communicate with the tumor would share antigenic specificity with their tumor-resident counterparts, making them an accessible means to monitor intratumoral immunity or to develop ex vivo cellular therapies. Also, responders to immunotherapy may exhibit posttreatment expansion of de novo specificities that were not present in the tumor at baseline and therefore may result from recruitment of circulating, non-exhausted cells from blood (9). To address these important questions, we conducted scRNA-seq and scTCR-seq from patients’ peripheral blood mononuclear cells (PBMCs), and analyzed this together with matched tumor and normal adjacent tissue (NAT) data from the same patients. Here, we identified cytotoxic CD4^+^ T cells with tumor-matching TCRs in blood that directly reflected the predominant antigenic specificities of intratumoral CD4^+^ T cells. These cells, in particular those that expressed granzyme B (GZMB), expressed high levels of the killer cell lectin-like receptor G1 (KLRG1), and proliferated in blood after PD-1 blockade. KLRG1 is an inhibitory receptor that identifies antigen-experienced T cells but has not been previously implicated in cytotoxic CD4^+^ function (10, 11). KLRG1 not only marks the majority of circulating cytotoxic tumor-TCR-matched CD4^+^ T cells, serving as a biomarker to monitor these cells as a window into intratumoral CD4^+^ T cell recognition, but also separately regulates their killing activity through its cognate interaction with tumor E-cadherin, as an inhibitory gate that operates independently of PD-1.

## Results

### CD4^+^ and CD8^+^ T cells in PBMCs and tumors contain conserved cytotoxic phenotypes.

**Figure 1:**
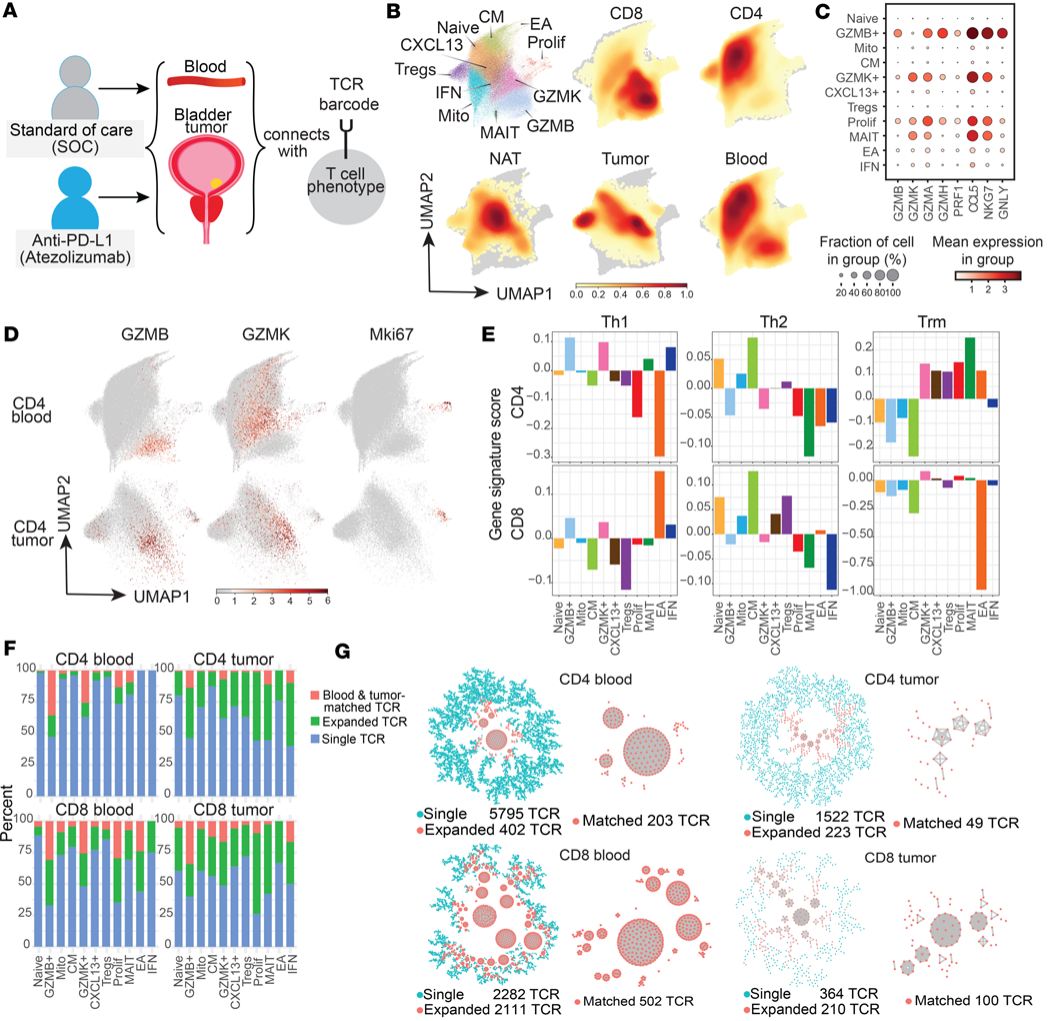
Combined blood and tumor TCR repertoire network analysis. (**A**) Blood and tumor samples were obtained from standard-of-care (SOC) and anti–PD-L1–treated patients with bladder cancer (on trial NCT02451423) for scRNA-seq/scTCR-seq, using matching TCRs on single cells as a barcode for matched antigenic specificity between compartments. (**B**) UMAP plot of 157,054 single cells clustered together, where each phenotypic population is identified with a distinct color and density plots showing distribution of cells for the subset of samples sorted for CD8^+^ or CD4^+^ T cells, or obtained from normal adjacent tissue (NAT), tumor, and blood. *n* = 7 tumors, 6 NAT, and 7 matched PBMC samples (before and after treatment for immunotherapy-treated patients). (**C**) Dot plot showing fractions of cells and mean expression of selected genes (bottom labels) in each cell cluster (side labels). (**D**) Feature plots showing expression of transcripts *GZMB*, *GZMK*, or *KI67* (red) in the cells from samples sorted for CD4^+^ cells (gray) from blood or tumor superimposed on the UMAP plot. CD4^+^ cells expressing cytotoxic transcripts are known to express CD4 protein based on sorting, but are coclustered with a predominance of CD8^+^ T cells due to their cytotoxic gene expression. (**E**) Phenotypic analysis of all CD4^+^ T cells using gene signatures for Th1, Th2, and Trm (pairwise comparison statistics in Supplemental Dataset 5). (**F**) Percentage of TCRs that are unique, expanded (cluster size >1), and expanded and blood-tumor matched for CD4^+^ and CD8^+^ T cells from blood and tumor. (**G**) Network TCR plots of a representative patient. Each dot represents 1 cell, and the dots within in each cluster in red have identical paired TCRαβ.

To determine whether T cell populations in blood reflected those within tumors of patients with muscle-invasive bladder cancer, CD4^+^ and CD8^+^ T cells were flow sorted from PBMCs from patients from whom tumor and NAT sequencing was previously obtained (7) (Figure 1A and Supplemental Dataset 1; supplemental material available online with this article; https://doi.org/10.1172/jci.insight.177373DS1). This included 3 patients treated with neoadjuvant anti–PD-L1 (atezolizumab) before cystectomy on a clinical trial (ClinicalTrials.gov NCT02451423), who had matched PBMC samples both before and after immunotherapy, along with their postimmunotherapy tumor data (Supplemental Dataset 1). We obtained scRNA-seq data from 124,467 PBMCs by 10× Genomics (12), and this dataset was combined with 32,587 single cells from tumors and NAT (7) before unbiased clustering with batch correction (13–15). There were 73,869 CD4^+^ T cells and 50,598 CD8^+^ T cells from PBMCs and 18,530 CD4^+^ T cells and 9,149 CD8^+^ T cells from tumors that passed filter.

Separately, we used the TCR from each single cell with RNA data to identify cells that were matched between blood and tumor, and their functional phenotype (Figure 1A). Using our published strategy to amplify TCR CDR3 sequences from 10× cDNA (7), paired TCR α and β (TCRαβ) sequences were obtained from 33,667 CD4^+^ T cells and 23,301 CD8^+^ T cells from PBMCs (45.6% and 46.1%, respectively, of all PBMC T cells with RNA data). These were combined with paired TCRs from 9,341 CD4^+^ and 4,935 CD8^+^ intratumoral T cells (50.4% and 53.9%, respectively, of all tumor T cells with RNA data) from the same patients (7).

Eleven T cell populations were identified. Differentially expressed (DE) genes were obtained for each population versus all other populations (Supplemental Dataset 2) and for individual CD4^+^ and CD8^+^ populations (Supplemental Dataset 3). Populations were annotated based on manual inspection of genes with the greatest differential expression (from largest cluster to smallest) as: Naive, *GZMB*^+^, mitochondria-rich (Mito), central memory (CM), *GZMK*^+^, C-X-C motif chemokine ligand 13^+^ (*CXCL13*^+^), regulatory T (Tregs), proliferative (Prolif), mucosal-associated invariant T (MAIT), early activated metabolic (EA), and IFN-pathway (IFN) cells. Uniform manifold approximation and projection (UMAP) (13) plots for these clusters are shown in Figure 1B. scRNA-seq from sorted CD4^+^ and CD8^+^ T cells from PBMCs, NAT, and tumors were combined for unbiased clustering in order to determine subsets that are similar or distinct against a common reference framework. Annotated cytotoxic CD4^+^ T cells still robustly grouped together when CD4^+^ and CD8^+^ T cells were initially clustered as separate populations (Supplemental Figure 1).

Four of 11 T cell populations were cytotoxic, as they overexpressed granzymes and cytotoxicity-associated genes such as *NKG7* and granulysin (*GNLY*) (Figure 1C). While these cytotoxic populations (described in detail below) comprised the majority of CD8^+^ T cells (mean ± SEM: 78.9% ± 2.4% and 59.5% ± 6.0% of intratumoral and circulating cells), they also constituted a meaningful fraction of CD4^+^ T cells as well (11.4% ± 0.7% and 10.2% ± 2.5% of intratumoral and circulating CD4^+^ cells). *GZMB* and *GZMK* overexpression was detected in distinct populations of CD4^+^ cells from blood as well as tumors of these patients (Figure 1D). In addition, both *GZMB*^+^ and *GZMK*^+^ CD4^+^ cells comprised a significant proportion of the *MKI67*^+^ proliferative population (Figure 1, C and D). Note that CD4^+^ T cells expressing cytotoxic transcripts (Figure 1D) were known to express CD4 protein based on our sort strategy, but co-clustered with CD8^+^ T cells due to their cytotoxic expression program (Figure 1B). MAIT cells had elevated expression of solute carrier family 4 member 10 (*SLC4A10*), killer cell lectin-like receptor B1 (*KLRB1*), and ZFP36 ring finger protein like 2 (*ZFP36L2*) (14), along with cytotoxic granzymes (*GZMA, GZMK*, and *GZMM*) (15). We previously observed proliferative cells expressing both markers of cytotoxicity (*GZMB* and *GZMK*) and proliferation (*MKI67*) in the tumor microenvironment (7). Similarly, our group and others have observed a cytotoxic population expressing several granzymes (*GZMA*, *GZMB*, *GZMH*, and *GZMM*), *GNLY*, and *PRF1* (perforin) but no *GZMK* (denoted as *GZMB*^+^), and a separate cytotoxic population that expressed *GZMK* that might express *GZMB* and *PRF1*, but not *GNLY* (denoted as *GZMK*^+^) (12, 16).

The remaining 7 T cell populations were noncytotoxic. *CXCL13*^+^ cells are potentially related to T follicular helper cells (Tfh), as they coexpressed PD-1 (*PDCD1*), inducible T cell costimulator (*ICOS*), and C-X-C motif chemokine receptor 5 (*CXCR5*) in CD4^+^ T cells (17). EA cells expressed cytochrome *c* (*CYCS*), hydroxyacyl-CoA dehydrogenase trifunctional multienzyme complex subunit α (*HADHA*), hypoxia-inducible factor 1 subunit α (*HIF1A*), and voltage-dependent anion-selective channel protein 1 (*VDAC1*). These markers were also recently observed in CD8^+^ T cells in response to *Listeria monocytogenes* infection in vivo, and in chimeric antigen receptor (CAR) T cells in patients with lymphoma (18). IFN pathway cells expressed IFN response genes such as IFN-induced protein with tetratricopeptide repeats 1 and 3 (*IFIT1* and *IFIT3*), MX dynamin-like GTPase 1 (*MX1*), and IFN regulatory factor 7 (*IRF7*) (19). Although IFN response genes are upregulated in response to viral infection (20) and may indicate immune priming in the cancer context, the function of this population in antitumor immunity has not been demonstrated. The Mito population marked by a significant proportion of mitochondrial genes (despite filtering out high mitochondrial gene expression >10%) was not characterized further as this may reflect cellular stress from tissue processing (21).

To further elucidate the function of each T cell population, we applied gene signatures (Supplemental Dataset 4) for Th1 (22), Th2 (22), and T tissue-resident memory (Trm) (23) (Figure 1E), in pairwise population comparisons (Supplemental Dataset 5). *GZMB^+^*, *GZMK^+^*, IFN, and MAIT populations displayed significantly higher Th1 scores versus other CD4^+^ populations. For CD8^+^ T cells, *GZMB^+^*, *GZMK^+^*, IFN, and EA populations exhibited higher Th1 scores. Conversely, cytotoxic CD4^+^ and CD8^+^ populations with Th1 characteristics (*GZMB*^+^, *GZMK*^+^, IFN) displayed significantly less Th2 phenotype versus other populations, while Naive, CM, and Treg populations had significantly higher Th2 scores. Finally, *GZMK*^+^, MAIT, Prolif, and *CXCL13^+^* CD4^+^ T cell populations had significantly higher Trm signatures than other CD4^+^ populations, suggesting propensity toward a tissue-resident phenotype, while *GZMB*^+^ cells did not.

### Circulating tumor-TCR-matching CD4^+^ T cells are predominantly cytotoxic.

Looking at the TCR repertoire of these cells, the percentage of total cells with a TCR that is unique, expanded (expressed by >1 cell), or expanded and matched between blood and tumor, is shown in Figure 1F for each phenotypic population. Additional information was obtained using TCR network analysis to cluster cells with the same TCR (24) (Figure 1G). Overall, 1,174 CD4^+^ T cells from PBMCs matched TCR clonotypes with 259 CD4^+^ T cells from tumors and 4,244 CD8^+^ T cells from PBMCs matched TCR clonotypes with 929 CD8^+^ T cells from tumors.

**Figure 2:**
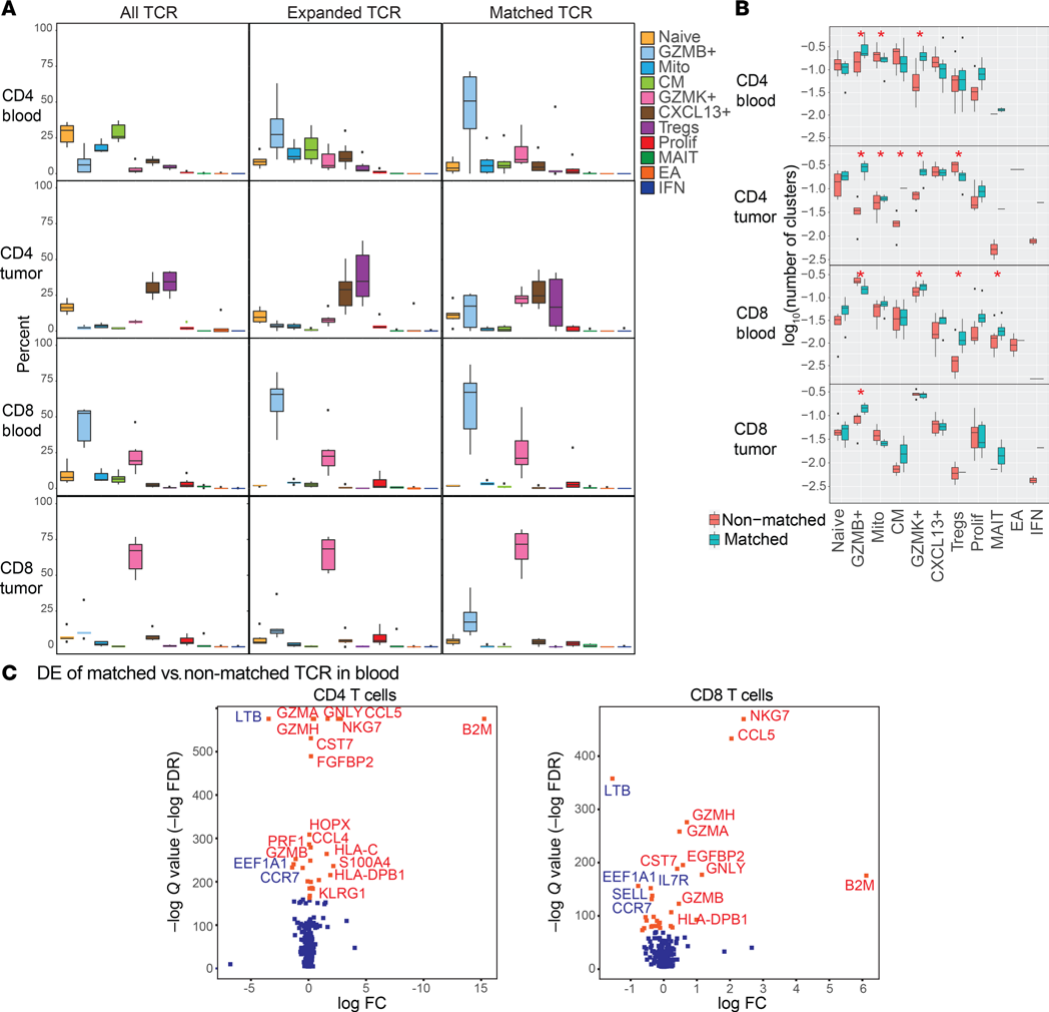
CD4^+^ and CD8^+^ T cells in PBMCs and tumor with matching TCRs are predominantly cytotoxic. (**A**) Box-and-whisker plots showing percentages of cells in each phenotypic population. Proportion of each cell phenotype within all TCRs, expanded TCRs, and blood- and tumor-matched TCRs in CD4^+^ and CD8^+^ T cells from blood and tumor. Statistical results of pairwise comparison for each population with the other populations are shown in Supplemental Dataset 6. *n* = 6 paired tumors and PBMCs. (**B**) Box-and-whisker plots with whiskers (median and interquartile ranges) showing number of TCR clusters containing each cell phenotype within matched and nonmatched TCRs in the blood. *FDR < 0.05. Linear mixed-model statistics are shown in Supplemental Dataset 7. *n* = 6 paired tumors and PBMCs. (**C**) Volcano plots showing DE genes of cells with matched TCRs versus cells with nonmatched TCRs from CD4^+^ and CD8^+^ T cells in the blood. Top genes by FDR are shown. Red highlights positive coefficient and blue negative coefficient. DE statistics in Supplemental Dataset 8.

Cytotoxic CD4^+^ phenotypes (i.e., *GZMB*^+^, *GZMK*^+^, Prolif which is largely cytotoxic, and MAIT) comprised the vast majority of CD4^+^ cells in blood that matched a TCR with tumor (Figure 2A). Specifically, the cytotoxic *GZMB*^+^ CD4^+^ cells (in blood), and *GZMB*^+^ and *GZMK*^+^ CD4^+^ cells (in tumor) were significantly enriched among CD4^+^ cells expressing a matched TCR, compared with total CD4^+^ cells where these cytotoxic phenotypes were not at all enriched (individual patient data Supplemental Figure 2, aggregate data Figure 2A, statistics Supplemental Dataset 6). In tumors, while blood-TCR-matched CD4^+^ cells also showed high frequencies of CXCL13^+^ and Tregs, this is because they were also prevalent among total CD4^+^ cells, indicating their basal contribution to the immune tumor microenvironment. Overall, *GZMB*^+^ cells comprised the majority (~50%) of circulating CD4^+^ cells with tumor-matched TCRs, and *GZMB*^+^ and *GZMK*^+^ cells together represented more than 40% of intratumoral CD4^+^ cells with blood-matched TCRs despite these cells constituting the minority of total CD4^+^ cells (Figure 2A). In contrast, *GZMB*^+^ and *GZMK*^+^ among CD8^+^ cells were highly abundant in blood regardless of TCR matching status (Figure 2A and Supplemental Dataset 6), reflecting that most CD8^+^ T cells are cytotoxic.

Quantitation using TCR network analysis (Figure 1G) further corroborated that cytotoxic CD4^+^ (*GZMB^+^* and *GZMK^+^*) T cells were significantly overrepresented in clusters of tumor-blood matched TCRs over nonmatched TCRs in blood and tumor (Figure 2B and Supplemental Dataset 7). Significant enrichment in tumor-blood matched TCR clusters was also seen in cytotoxic CD8^+^ (*GZMK*^+^, *GZMB*^+^, and MAIT) T cells (Figure 2B). Taken together, these analyses of matched TCRαβ sequences, which are directly clonally related, indicate that cytotoxic CD4^+^ tumor-infiltrating lymphocytes (TILs) from tumors communicate with the circulation. Circulating cytotoxic CD4^+^ are the majority of the CD4^+^ cells in blood that shared specificity with tumors. Hence, the identification and isolation of cytotoxic CD4^+^ T cells in blood provides a direct window into the specificity of intratumoral CD4^+^ T cells.

We further examined the function of circulating cells with tumor-matching TCRs, using DE analysis to compare circulating cells with tumor-matching versus nonmatched TCRs (Figure 2C and Supplemental Dataset 8). Genes that were overexpressed (*P* < 0.01) in CD4^+^ cells in blood were cytotoxic genes *GZMA, GZMH, GZMB, GNLY, PRF*, and *GZMK*; the chemokines C-C motif chemokine ligand 4 and 5 (*CCL4* and *CCL5*); and *KLRG1*, which is associated with effector T cell phenotypes and also has regulatory capacity for immune cells. Furthermore, KLRG1 may mark circulating cytotoxic CD4^+^ T cells with tumor-matched TCRs to allow their isolation, which we validate below. Other enriched genes in blood- and tumor-TCR-matched populations included antigen presentation (β-2-microglobulin [*B2M*] and human leukocyte antigens [HLAs] for both MHC class I and class II). Conversely, TCR-matched circulating CD4^+^ T cell populations downregulated lymphotoxin β (*LTB*) and genes associated with the naive phenotype such as C-C motif chemokine receptor 7 (*CCR7*), L-selectin (*SELL*), and IL-7 receptor (*IL7R*), supporting an effector phenotype of these cells. Similar DE genes were observed for tumor-TCR-matched, circulating CD8^+^ T cells.

### Circulating tumor-TCR-matched cytotoxic CD4^+^ T cells proliferate after PD-1 blockade.

**Figure 3:**
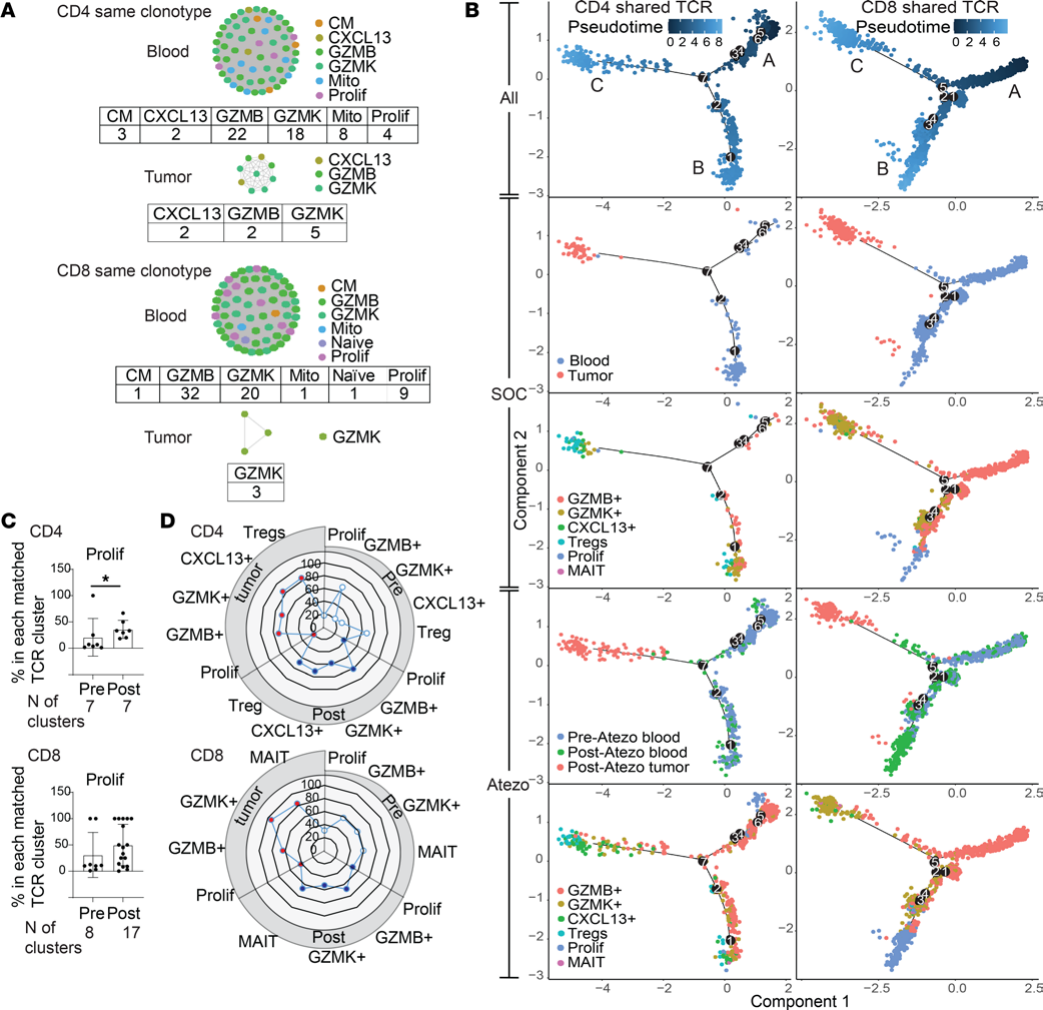
Phenotypic heterogeneity and plasticity of individual TCR clusters. (**A**) A representative CD4^+^ T cell network cluster with matching TCR clonotype from blood and tumor, and a separate CD8^+^ T cell network cluster with a different matching TCR clonotype. Each dot is 1 cell and the color represents a different cellular phenotype. Tables show the number of cells with the indicated phenotype in the cluster. (**B**) Pseudotime trajectories of GZMB^+^, GZMK^+^, CXCL13^+^, Tregs, Prolif, and MAIT cells with blood- and tumor-matched TCRs in CD4^+^ and CD8^+^ T cells from 2 untreated standard-of-care (SOC) patients and 3 patients treated with anti–PD-L1 (atezolizumab, Atezo) on trial. Black circles are branch nodes. Major branches A, B, and C are labeled. (**C**) Plots showing percentage of Prolif cell phenotype in each TCR cluster from blood- and tumor-TCR-matched clusters in blood before and after anti–PD-L1 treatment. The number (*n*) of clusters is listed below the plots. *P < 0.05 by nonparametric Mann-Whitney *U* test. (**D**) Radar plots of mean percentage of selected cell phenotype in each TCR cluster in matched TCR clusters of pretreatment PBMCs (white circles), post-Atezo-treatment PBMCs (blue circles), and post-Atezo-treatment tumors (red circles) for CD4^+^ and CD8^+^ T cells.

TCR network analysis also revealed that cells with a matching TCR clonotype in a cluster that share identical antigen specificity could have different phenotypes (Figure 3A). Since the TCRs within each cluster have exact amino acid matches of paired, genomically rearranged TCRαβ CDR3 sequences, these cells likely did not arise from convergent evolution of different T cell clones, and instead likely originated from a single parent cell. These plots provide examples of related developmental subsets of CD4^+^ T cells that are linked by coexpression of the same TCRαβ. Also, exact TCR matching of clones in blood and tumor indicates their circulation between compartments. In order to infer fate trajectories of these different phenotypes that were clonally linked by a common TCR barcode, pseudotime analysis by Monocle was carried out specifically on cells with tumor-blood-matched TCRs from *GZMB*^+^, *GZMK*^+^, *CXCL13*^+^, Tregs, Prolif, and MAIT populations (Figure 3B).

To assess baseline developmental trajectories in bladder cancer, and how these are affected by immunotherapy, we performed pseudotime analysis on blood and tumors from the 2 standard-of-care patients with bladder cancer who did not receive any preoperative systemic therapy (Untreated A and B), together with the 3 trial patients treated with atezolizumab (Anti-PD-L1 B–D, who had blood both pre- and posttreatment, and posttreatment tumor; Supplemental Dataset 1), and examined results from these groups separately. The proportion of each cell phenotype in the 3 major pseudotime branches (A, B, and C) was quantitated per sample before and after immunotherapy (Supplemental Dataset 9). Given the limited sample size, this analysis identifies descriptive trends influenced by immunotherapy. Branches A and B contained mostly cells from blood, and branch C contained predominantly cells from tumors (Figure 3B). In blood, before PD-1 blockade, tumor-TCR-matched CD4^+^ cells were predominantly GZMB^+^ in pre-atezolizumab blood (average 35%–37% of branches A and B), with a minority of GZMK^+^ (2.8%–5.2% of branches A and B). After PD-1 blockade, cytotoxic proliferation increased in paired post-atezolizumab blood compared with pre-atezolizumab blood (6.5% vs. 0.8% in branch A, 0.7% vs. 0% in branch B; Figure 3B, left column). Peripheral mobilization of cytotoxic CD4^+^ T cells by PD-1 blockade is also suggested by the new appearance of GZMB^+^ cells in post-atezolizumab blood (4.2%, with 0% pre-atezolizumab) in the same branch C that contains most of the intratumoral T cells, suggesting a T cell phenotype transitioning to the tumor. In the tumor, PD-1 blockade led to more GZMB^+^ and CXCL13^+^ CD4^+^ cells, and fewer Tregs, in post-atezolizumab tumors versus untreated standard-of-care tumors (22% vs. 0%, 40% vs. 21%, 12% vs. 50% in branch C; Figure 3B, left column), supporting intratumoral mobilization of cytotoxic CD4^+^ effectors. Intratumoral GZMK^+^ cells were abundant but unchanged by immunotherapy treatment for CD4^+^ (24% in post-atezolizumab tumors vs. 25% in untreated standard-of-care tumors, branch C) and CD8^+^ (76% vs. 73%). While CD8^+^ cells from the same samples showed similar proliferation in post-atezolizumab blood versus pre-atezolizumab blood (24% vs. 1.6%; Figure 3B, right column, branch A), the composition of intratumoral CD8^+^ cell types remained mostly similar comparing post-atezolizumab versus untreated standard-of-care tumors (Figure 3B, right column, branch C). This indicates that any immunotherapy-induced differences seen in cytotoxic CD4^+^ T cell proportions were specific to these cells, and were not due to sampling variability.

We could also quantitatively assess the functional subtypes within each tumor-matching TCR cluster before and after anti–PD-L1 treatment. The composition of TCR clusters in blood shifted with PD-1 blockade to a higher proportion of proliferating cytotoxic cells, suggesting that these cells may act as progenitors in blood, whose early mobilization with immunotherapy may give rise to other cytotoxic phenotypes (Figure 3C). A similar trend was observed for circulating CD8^+^ T cells, although it was not significant. Within tumor, blood-matched TCR phenotypes were predominantly not proliferating, but were instead consistently cytotoxic (*GZMB*^+^ and *GZMK*^+^ for CD4^+^, also *GZMK*^+^ and MAIT for CD8^+^), and also included noncytotoxic *CXCL13*^+^ CD4^+^ and Tregs as observed above (Figure 3D).

### Proliferative cytotoxic CD4^+^ T cells upregulate PD-1 and Tim-3, and downregulate KLRG1, as a consequence of their activation.

**Figure 4:**
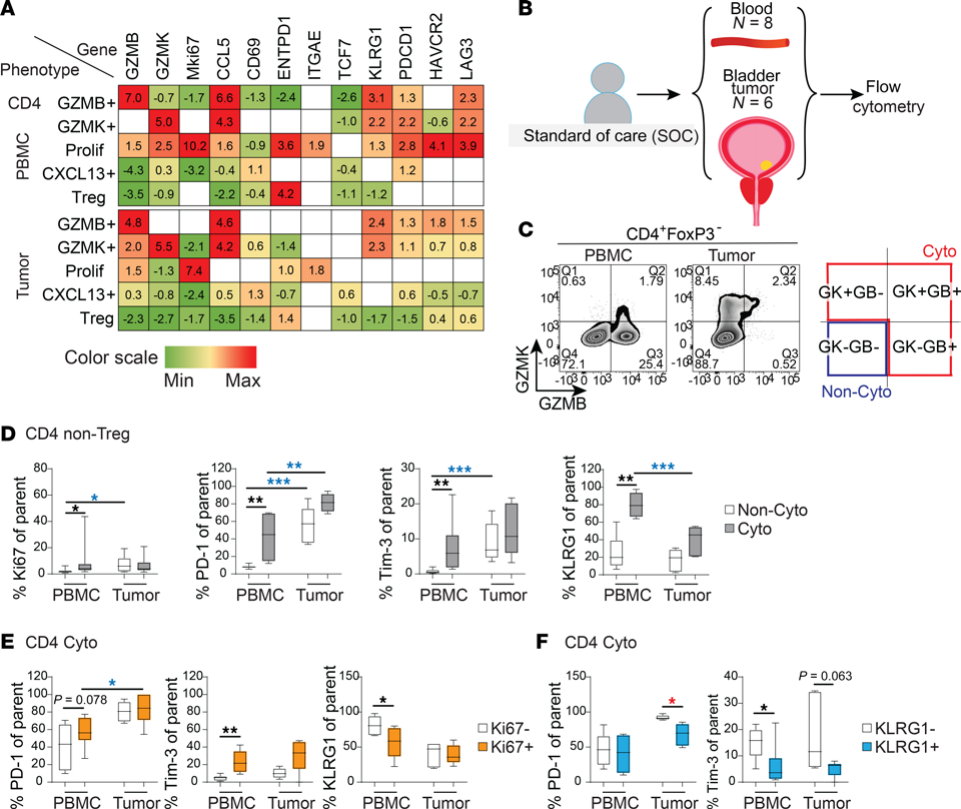
Expression of proliferative and inhibitory markers on cytotoxic versus noncytotoxic cell types in PBMCs and bladder tumors. (**A**) Normalized log(fold change) from scRNA-seq analysis for selected phenotypes and genes. (**B**) Flow cytometry was carried out on 8 PBMC samples and 6 tumors from standard-of-care (SOC) patients with bladder cancer. (**C**) Representative flow cytometry plots showing CD4^+^FoxP3^–^ T cells expressing GZMB and GZMK, and total cytotoxic subtypes (Cyto) and noncytotoxic subtypes (Non-cyto) subtypes are gated as shown. (**D**) Box-and-whisker plots showing percentage Ki67^+^, PD-1^+^, Tim-3^+^, and KLRG1^+^ of Cyto and Non-Cyto from CD4^+^FoxP3^–^ T cells. (**E**) Box-and-whisker plots showing percentage PD-1^+^, Tim-3^+^, and KLRG1^+^ of Ki67^+^ and Ki67^–^cytotoxic cells. (**F**) Box-and-whisker plots showing percentage PD-1^+^ and Tim-3^+^ of KLRG1^+^ and KLRG1^–^ cytotoxic cells. Comparison of paired cell subsets within PBMCs or tumors was performed using Friedman’s test with Dunn’s multiple-comparison test. Comparison of nonpaired cell subsets between PBMCs and tumors was performed using Kruskal-Wallis test with Dunn’s multiple-comparison test. *P < 0.05; **P < 0.01; ***P < 0.001. Asterisks in black, red, or blue indicate significant differences between subsets within PBMCs, within tumors, and between PBMCs and tumors, respectively. Corresponding plots for CD8^+^ cells are shown in Supplemental Figure 4.

Looking more closely at these circulating cytotoxic and proliferating cytotoxic CD4^+^ phenotypes, we noticed that blood GZMB^+^ and GZMK^+^ CD4^+^ T cells expressed high levels of *KLRG1* by scRNA-seq (Figure 4A, data extracted from Supplemental Dataset 3, and also previously noted in Figure 2C), as well as moderate levels of *PDCD1* and *LAG3* (Figure 4A). Their proliferating CD4^+^ counterparts upregulated *ENTPD1* (CD39) and *ITGAE* (CD103) (Figure 4A), like proliferating CD8^+^ T cells (Supplemental Figure 4A), and hence this mobilized population is enriched for putative tumor antigen specificity (25–27). This motivated further study of the proliferating cytotoxic CD4^+^ population and its regulation, as notably, these proliferating cells actually downregulated *KLRG1* expression while further upregulating *PDCD1*, *LAG3*, and *HAVCR2* (Tim-3) (Figure 4A for CD4^+^, Supplemental Figure 4A for CD8^+^). While upregulation of these immune checkpoints might be regulatory, it could result from exhaustion or activation.

To validate and investigate the relationship between these surface immune checkpoints at baseline in untreated standard-of-care patients with cancer, we performed flow cytometry on blood from 8 patients with localized bladder cancer, 3 of whom had matched tumors and 3 additional nonmatched tumors (Figure 4B). These patients were completely distinct from those used for scRNA-seq, allowing us to validate the surface phenotype of cytotoxic CD4^+^ T cells. For initial analysis, while we gated cytotoxic populations separately based on distinct expression of GZMB and/or GZMK, we pooled all granzyme^+^ cells as a larger cytotoxic population and compared them to their granzyme^–^ noncytotoxic counterparts (Figure 4C, gating strategy in Supplemental Figure 3). In the periphery, the cytotoxic CD4^+^ fraction demonstrated significantly more Ki67 protein expression, as well as the checkpoints PD-1, Tim-3, and KLRG1, compared with noncytotoxic cells (Figure 4D). The high frequency of KLRG1 expression is noteworthy, as it was found in approximately 80% of cytotoxic CD4^+^ T cells, identifying this as a surface marker allowing isolation of this population and its enriched repertoire of tumor-shared clones. These patterns of proliferation and checkpoint expression are consistent with scRNA-seq and indicate that circulating cytotoxic CD4^+^ T cells are uniquely enriched for proliferative capacity and immune checkpoint expression compared with their noncytotoxic counterparts. This is in contrast to tumor, where a much higher proportion of both cytotoxic and noncytotoxic CD4^+^ T cells globally expressed PD-1 and Tim-3 (which may be related to exhaustion), but lower levels of KLRG1, suggesting distinct mechanisms of regulation from blood. These observations were similarly found for CD8^+^ T cells (Supplemental Figure 4, B–D).

Closer examination reveals divergent relationships between proliferation and expression of either conventional immune checkpoints or KLRG1 in cytotoxic CD4^+^ T cells. Specifically, when we divide cytotoxic CD4^+^ T cells into proliferating and nonproliferating groups, in blood the proliferating Ki67^+^ cytotoxic CD4^+^ T cells trended toward higher PD-1, and had significantly higher Tim-3 expression (Figure 4E). Conversely, proliferating cytotoxic CD4^+^ T cells significantly downregulated KLRG1 (Figure 4E), indicating a distinct pattern of expression in mobilized cells and suggesting that KLRG1’s regulatory role for cytotoxic CD4^+^ T cells is distinct from PD-1 and Tim-3. In particular, the fact that the vast majority of total circulating cytotoxic CD4^+^ T cells expressed KLRG1 (Figure 4D) indicates that their basal nonactivated state is linked to high KLRG1, while their activation and proliferation are accompanied by concomitant downregulation of KLRG1 and upregulation of Tim-3 (Figure 4F). These distinct patterns of regulation by KLRG1 compared with immune checkpoints were also seen in cytotoxic CD8^+^ T cells (Supplemental Figure 4, E and F).

### Circulating GZMB^+^ CD4^+^ T cells are naive-like or effectors, while GZMK^+^ CD4^+^ T cells adopt a memory phenotype.

**Figure 5:**
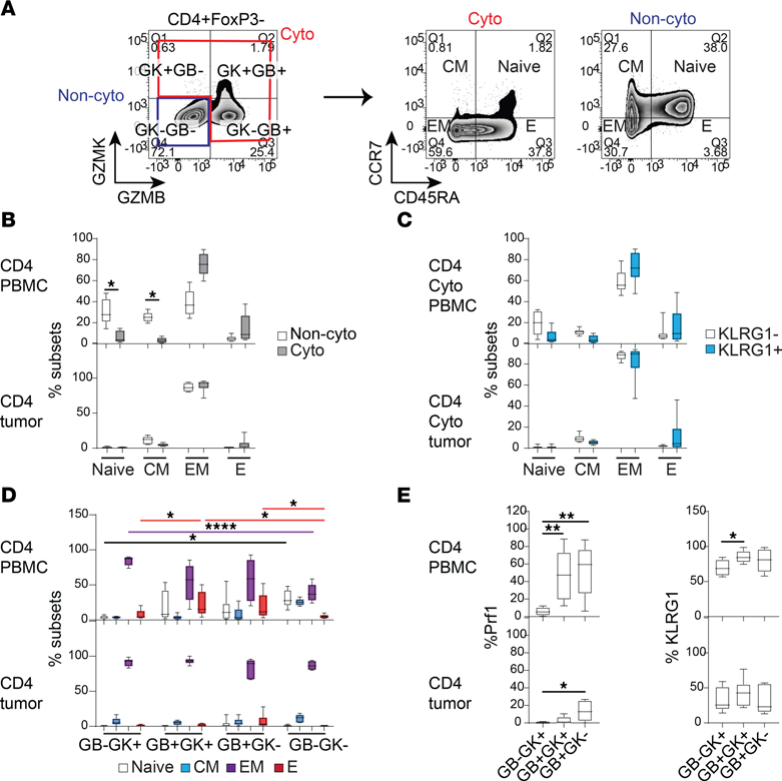
Developmental stages of different cytotoxic cell types in PBMCs and bladder tumors. (**A**) Flow cytometry was carried out on 8 PBMC samples and 6 tumors from standard-of-care patients with bladder cancer. Representative flow cytometry plots showing CD4^+^FoxP3^–^ T cells expressing GZMB and GZMK, and Cyto and Non-Cyto cells were further gated into Naive, CM, EM, and E based on expression of CCR7 and CD45RA. All graphs in this figure are box-and-whisker plots for CD4^+^ T cells from PBMCs and tumors showing (**B**) proportion of developmental subsets in Cyto and Non-cyto cells; (**C**) proportion of developmental subsets in KLRG1^–^ and KLRG1^+^ cytotoxic cells; (**D**) proportion of developmental subsets in each cytotoxic subtype (red, purple, and black lines indicate significant pairwise comparison of proportion of E, EM, or Naive subsets, respectively, between cytotoxic subtypes); and (**E**) percentage perforin^+^ (Prf1^+^) and KLRG1^+^ of each cytotoxic subtype. Comparison of paired cell subsets was performed using Friedman’s test with Dunn’s multiple-comparison test. *P < 0.05; **P < 0.01; ****P < 0.0001. Corresponding plots for CD8^+^ cells are shown in Supplemental Figure 4.

Although the proliferative tumor-matching cytotoxic CD4^+^ T cells in blood are of interest for their putative tumor recognition, how they map onto specific GZMB- or GZMK-expressing subsets, and more generally the ontological relationship between distinct granzyme-expressing populations, has not been explored. We first examined this in the distinct standard-of-care patients with bladder cancer not treated with immunotherapy as above, to understand basal relationships. In addition to separating cytotoxic subtypes by expression of GZMB alone (GB^+^GK^–^), GZMK alone (GB^–^GK^+^), or GZMB and GZMK together (GB^+^GK^+^), we also subdivided total cytotoxic cells into Naive, CM, Effector Memory (EM), and Effector (E) defined by CCR7 and CD45RA protein expression (Figure 5A). Of note, as GZMB and GZMK are effector molecules, their expression in cells classified as “Naive” indicates these are actually “Naive-like.” Total cytotoxic CD4^+^ T cells were predominantly EM and E in phenotype in both blood and tumor (Figure 5B). Although KLRG1 was also largely expressed by EM and E cytotoxic CD4^+^ T cells, consistent with its typical expression pattern (28), KLRG1^+^ cytotoxic CD4^+^ T cells did not demonstrate a difference in effector or memory frequency compared to KLRG1^–^ cells (Figure 5C). Looking at developmental status by granzymes, a key difference was that GZMB-expressing cells (GB^+^GK^+^ or GB^+^GK^–^) contained more “Naive-like” and E phenotypes, while the solely GZMK-expressing cells (GB^–^GK^+^) were almost entirely EM, with a significantly higher EM proportion compared with noncytotoxic cells. These differences were observed only in blood and not in tumor (Figure 5D). This distinction in GZMB- versus GZMK-associated phenotypes was substantiated by significantly higher perforin protein expression in GZMB-expressing subsets, and higher KLRG1 expression at least in the GB^+^GK^+^ subset in blood, consistent with an effector phenotype (Figure 5E). These findings support a dichotomy in development of cytotoxic CD4^+^ subsets in blood occurring in the untreated steady state: GZMB-expressing cells are the more cytolytically active effector cells and have higher expression of the regulatory receptor KLRG1, which may check their activity in blood. As a consequence, these GZMB^+^ cells were not proliferating in the periphery without treatment. On the other hand, cytotoxic CD4^+^ cells that solely expressed GZMK did not coexpress perforin and were less cytolytic, and may represent a memory phenotype that may be less regulated by KLRG1. Similar patterns of perforin expression were seen also for cytotoxic CD8^+^ T cells in untreated patients, except their GZMB-only expressing cells were less regulated by KLRG1 in tumor (Supplemental Figure 4, G and H).

### PD-1 blockade induced proliferation of GZMB-expressing circulating cytotoxic CD4^+^ T cells.

**Figure 6:**
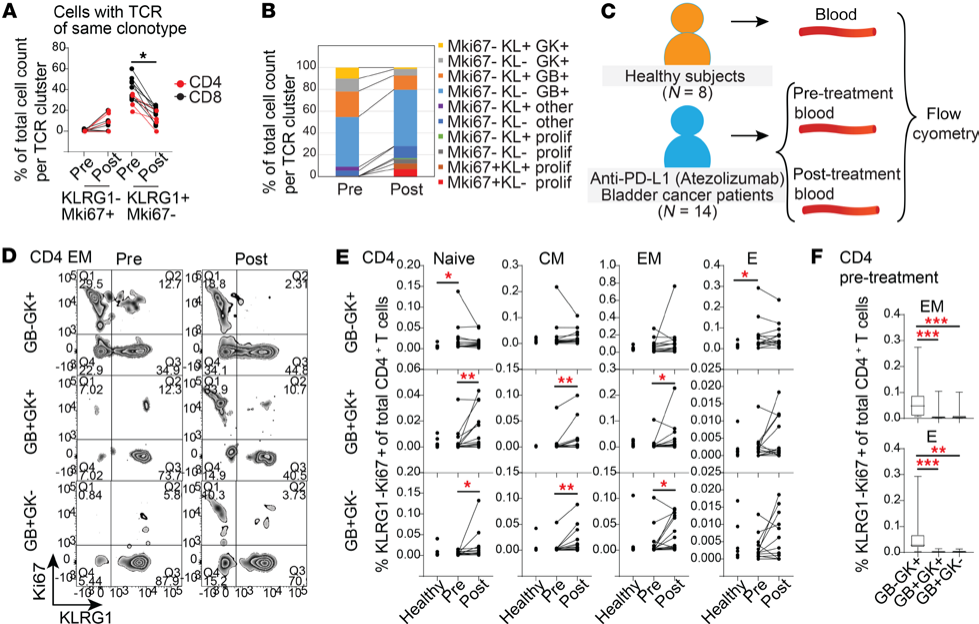
Anti–PD-L1 treatment increased Ki67 and decreased KLRG1 expression on cytotoxic T cells in the blood. (**A**) Percentage of cells expressing either *Mki67* or *Klrg1* transcript in each TCR cluster from pre- and post-atezolizumab patient PBMCs. Each line represents a different TCR cluster. Friedman’s test with Dunn’s multiple-comparison test was carried out pre- and post-treatment timepoints for CD4^+^ (red) and CD8^+^ (black) TCR clusters (TCR clusters = 13). (**B**) Stacked bar plot showing mean percentage of cells expressing *Mki67* and/or *Klrg1* (KL) transcripts before and after anti–PD-L1 treatment, as shown per TCR cluster (TCR clusters = 13). (**C**) Flow cytometry was carried out on PBMCs from 8 healthy individuals and 14 pre- and post-atezolizumab-treated patients with bladder cancer. (**D**) Representative flow cytometry of pre- and posttreatment CD4^+^ EM cytotoxic T cells showing Ki67 and KLRG1 expression. (**E**) Percentage KLRG1^–^Ki67^+^ among total CD4^+^ T cells in cytotoxic subtypes with Naive-like, CM, EM, and E phenotypes. (**F**) Comparisons of pretreatment percentage of KLRG1^–^Ki67^+^ among total CD4^+^ T cells between cytotoxic subsets. Mann-Whitney *U* test was used to compare healthy and pretreatment samples; Wilcoxon’s matched-pairs signed-rank *t* test was used to compare pre- and posttreatment samples. *P < 0.05; **P < 0.01; ***P < 0.001. Corresponding plots for CD8^+^ cells are in Supplemental Figure 6.

To examine immunotherapy-induced changes in proliferative cytotoxic CD4^+^ T cells, and how this relates to regulatory KLRG1 expression, we first examined them in our prior network single-cell analysis from the 3 trial patients with paired tumor and longitudinal blood (both before and after immunotherapy). As KLRG1 downregulation accompanied increased proliferation in the steady state in standard-of-care patients with bladder cancer (Figure 4E), we looked at how PD-1 blockade affected coregulation of KLRG1 and proliferative capacity in clonally related cells. Within each matched TCR cluster (i.e., single cells with the same TCRαβ), we quantitated the proportion of cells containing *KLRG1* and *MKI67* transcripts before and after anti–PD-L1 treatment, within a population where we could track clonal relationships between progenitor and progeny cells. Per TCR cluster, the proportion of nonproliferative cells that were *KLRG1*^+^ but *MKI67*^–^ significantly decreased with immunotherapy treatment (across multiple granzyme-expressing subtypes), while proliferative cells that were either *KLRG1*^–^ *MKI67*^+^ or *KLRG1*^+^ *MKI67*^+^ increased after treatment (Figure 6, A and B). The proportion of cells that were negative for *KLRG1* and *MKI67* transcripts was also higher after treatment for *GZMB*^+^ and Prolif cells. This could be due to downregulation of *KLRG1* without proliferation, or a proliferative phenotype where we cannot detect *MKI67*. Hence, PD-1 blockade enhanced concomitant KLRG1 downregulation and enhanced proliferation from GZMB- and GZMK-expressing cytotoxic CD4^+^ and CD8^+^ T cells.

We next performed flow cytometry on paired pre- and posttreatment PBMCs from 14 patients with localized bladder cancer treated with neoadjuvant anti–PD-L1 before cystectomy on trial NCT02451423 to validate cytotoxic CD4^+^ T cells and their developmental and functional transitions with immunotherapy at the protein level. While 4 of these trial patients had overlapping tumor, and 4 patients had overlapping blood, with our scRNA-seq dataset, the other 10 trial patients were completely distinct from the scRNA-seq (Figure 6C, gating strategy in Supplemental Figure 5). As our prior analysis for standard-of-care patients, we similarly divided cytotoxic subtypes into developmental stages by CCR7 and CD45RA. PD-1 blockade increased the frequency of proliferative, KLRG1^–^Ki67^+^CD4^+^ T cells in blood that expressed GZMB (i.e., GB^+^GK^+^ and GB^+^GK^–^subsets) out of total CD4^+^ T cells. This was seen across Naive-like, CM, and EM populations (Figure 6, D and E). In contrast, solely GZMK-expressing CD4^+^ T cells that lacked GZMB were more proliferative before treatment compared with healthy controls (Figure 6D), particularly in Naive and E populations (Figure 6E), but this was not significantly increased by PD-1 blockade (Figure 6, D and E). The baseline proliferative capacity of GZMK^+^CD4^+^ T cells was also significantly higher than other, GZMB-expressing subtypes (Figure 6F). Together, these results suggest that across developmental stages, GZMB-expressing cytotoxic CD4^+^ T cells do not proliferate at baseline but are newly mobilized by PD-1 blockade. In contrast, basal proliferation of GZMK^+^CD4^+^ T cells in the periphery before treatment in patients with cancer suggests chronic activation stemming from the presence of tumor. As GZMB^+^ and GZMK^+^ states were directly interrelated by their expression of matching TCRs for common antigens, GZMB^+^CD4^+^ T cells may represent newly activated cells, which later give rise to the chronically activated GZMK^+^ population. Their CD8^+^ counterparts showed similar mobilization after PD-1 blockade of KLRG1^–^Ki67^+^ proliferating GZMB-expressing cells across CM, EM, and E status (Supplemental Figure 6, A–C); however, a key difference from cytotoxic CD4^+^ is the behavior of GZMK^+^CD8^+^ T cells, which do not demonstrate baseline proliferation over healthy controls or the GZMB-expressing CD8^+^ subsets.

### Cytotoxicity of circulating KLRG1^+^CD4^+^ T cells is restricted by E-cadherin.

**Figure 7:**
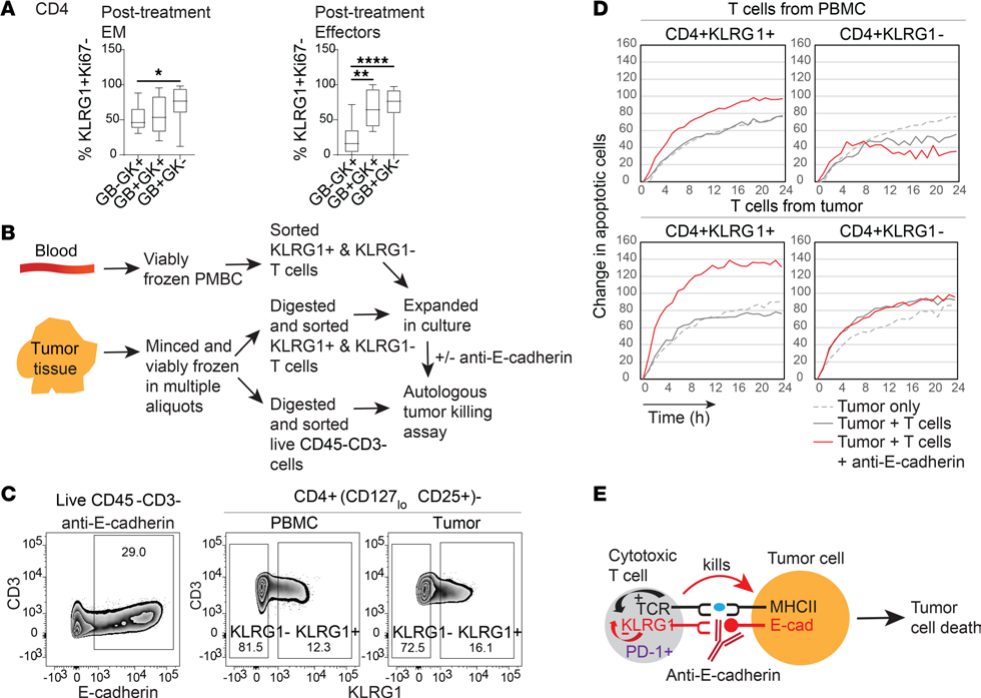
Expression and function of KLRG1 in cytotoxic CD4^+^ T cells. (**A**) Box-and-whisker plot showing percentage of KLRG1^+^Ki67^–^ T cells among 14 post-atezolizumab-treatment EM and E cytotoxic subsets in PBMCs. Friedman’s test with Dunn’s multiple-comparison test was carried out. *P < 0.05; **P < 0.01; ****P < 0.0001. (**B**) Strategy for sorting and autologous tumor killing assay. (**C**) Flow cytometry of E-cadherin staining of live CD45^–^CD3^–^ cells in a portion of tumors used in the killing assay, and KLRG1^+^ and KLRG1^–^ gates of CD4^+^(CD127^lo^CD25^+^)^–^ cells from PBMCs and tumors sorted for T cell expansion. (**D**) Apoptotic tumor cell death plotted as the relative change in annexin V^+^ cell count from time zero, with background T cell death subtracted at each time point. Results for tumor 1 are shown (results for tumors 2 and 3 in Supplemental Figure 7). (**E**) Model of inhibition of cytotoxic T cells by binding of KLRG1 on T cells to E-cadherin on tumor cells. Inhibition of cytotoxic cells by KLRG1 is alleviated by blocking with an anti–E-cadherin antibody.

E-cadherin is a known ligand of KLRG1 that gates KLRG1’s inhibitory activity across T cell subtypes (29). However, although we found enrichment of KLRG1 on circulating tumor-TCR-matching cytotoxic CD4^+^ T cells, the functional relevance of this ligand-receptor interaction for tumor recognition and killing in this subset has not been studied. We hypothesized that the activity of cytotoxic CD4^+^ T cells is gated by engagement of KLRG1 by E-cadherin on tumor cells via direct interaction. This is based on several findings. In both untreated and immunotherapy-treated patients with bladder cancer, proliferation of circulating cytotoxic CD4^+^ T cells was accompanied by downregulation of KLRG1. Furthermore, although we demonstrated that PD-1 blockade alone can cause proliferation in a limited subset of GZMB-expressing cytotoxic CD4^+^ T cells, there were still significant posttreatment levels of nonproliferative GB^+^GK^+^ and GB^+^GK^–^ cells that expressed KLRG1 in validation trial patients largely distinct from our scRNA-seq data (Figure 7A). This is also the case for cytotoxic CD8^+^ T cells (Supplemental Figure 7A). This latter observation suggests that adding KLRG1 blockade to PD-1 blockade can further mobilize additional tumor-TCR-matching cytotoxic CD4^+^ T cells in the periphery to kill.

To test this, we expanded KLRG1^+^ and KLRG1^–^ T cells from PBMCs and tumors of the same patient, coincubated with autologous tumor cells, and measured tumor cell killing (Figure 7B). Primary tumors in these experiments expressed E-cadherin protein, and the circulating and intratumoral non-Treg CD4^+^ cells expressed KLRG1 (Figure 7C). It is not possible to sort prospectively for granzyme-expressing cells prior to live-cell killing, as granzyme staining is intracellular and would require cell permeabilization. However, flow cytometry on the culture-expanded patient CD4^+^ T cells used in our killing assay found nearly 100% of the KLRG1^+^CD4^+^ T cells expressing GZMB and/or GZMK (Supplemental Figure 7B). For the first tumor, neither KLRG1^–^ nor KLRG1^+^ CD4^+^ T cells from PBMCs increased tumor cell death when coincubated without treatment. However, adding anti–E-cadherin increased tumor cell death, which was specifically seen with KLRG1^+^ cells but not KLRG1^–^cells (Figure 7D, top). KLRG1 played a similar role for cytotoxic CD4^+^ T cells from tumors. While KLRG1^–^CD4^+^ T cells have some spontaneous killing activity due to the presence of tumor-specific T cells that are not inhibited by KLRG1, E-cadherin blockade did not enhance their killing. Blocking E-cadherin did instead enhance tumoricidal activity of KLRG1^+^ intratumoral CD4^+^ T cells (Figure 7D, bottom). Similar findings were obtained in 2 additional tumors. In a separate patient, we confirmed enhancement of tumor killing by circulating and intratumoral CD4^+^ T cells by E-cadherin blockade that is specific for KLRG1, and also MHC class II in agreement with our prior findings (7), and was not impacted by isotype control antibodies (Supplemental Figure 7C). We also confirmed that the observed death from CD4^+^ T cell coculture was from tumors (using directly labeled tumor target cells) and not T cells, and that E-cadherin blockade can also enhance CD8^+^ T cell tumor killing in a KLRG1-dependent manner despite some spontaneous killing activity (Supplemental Figure 7D). Thus, functional studies across 3 unique patients, distinct from scRNA-seq or flow cytometry cohorts, validate that KLRG1–E-cadherin interactions can inhibit MHC class II–dependent autologous tumor killing by cytotoxic CD4^+^ T cells (Figure 7E), in a mechanism that is distinct from and complements PD-1 blockade. Additionally, we have also obtained single-cell TCRαβ sequences from expanded KLRG1^+^ and KLRG1^–^ CD4^+^ T cells from PBMCs, and matched total CD4^+^ T cells from tumor, of 2 patients. This showed that KLRG1^+^ sorted cells have a higher proportion of tumor-matching TCRs compared with KLRG1^–^ sorted cells (Supplemental Dataset 10).

## Discussion

There is growing appreciation for cytotoxic effector cells other than conventional CD8^+^ T cells that can recognize and kill tumors, and can be therapeutically mobilized to expand the benefit of cancer immunotherapy. In particular, while cytotoxic CD4^+^ T cells have long been observed in tumor models and patients with cancer, recent work has highlighted their functional importance in recognizing MHC class II–restricted tumor antigens, and their regulation in vivo in patients (1, 4, 6–8, 30). However, while these cytotoxic CD4^+^ T cells have also been observed in the blood of patients with cancer, the origin and functional relevance of this circulating subset and how their activity is regulated in the periphery has remained obscure.

By sorting for CD4 and CD8 proteins prior to single-cell sequencing, we were able to identify cytotoxic CD4^+^ T cells and compare them to CD8^+^ T cells. If not sorted for coreceptor expression, these cells’ low expression of *CD4* RNA is not enough to separate them, as otherwise their transcriptional signature is dominated by cytotoxicity genes, similar to CD8^+^ T cells. Hence, their study in unsorted scRNA-seq from patients with cancer requires specific isolation of *CD4*-expressing cytotoxic cells, which was done in reanalysis of published data to provide evidence for cytotoxic CD4^+^ T cells in breast cancer, head and neck cancer, melanoma, and liver cancer (8).

Additionally, by scrutinizing cells that share TCRαβ in paired blood and tumors, and tracking their phenotype, we identified cytotoxic CD4^+^ T cells with matching TCRs in blood and tumor. This supports circulation of these cells between compartments. Our further studies provide several insights that we believe to be novel regarding the ontogeny and regulation of these cells, and diagnostic and therapeutic applications for immunotherapy.

First, cytotoxic CD4^+^ T cells are the dominant CD4^+^ population in blood, sharing specificity with tumor-resident CD4^+^ cells despite being the minority of total CD4^+^ cells. Hence, the study of these accessible cells in blood provides a broader representation of the antigenic specificities of intratumoral CD4^+^ T cells, including those that recognize tumor antigens. This has direct implications for development of cellular therapies that prospectively isolate and expand this population. Furthermore, KLRG1 is expressed on at least 80% of these tumor-shared CD4^+^ T cell clones in the periphery, and KLRG1^+^ blood populations are highly enriched for tumor-matching clones in ex vivo–expanded CD4^+^ T cells from paired patient blood and tumors. Thus, KLRG1 is a diagnostic marker for isolation and further study of tumor-matching, cytotoxic CD4^+^ T cell clones from the periphery of patients with cancer.

Second, proliferation of cytotoxic CD4^+^ T cells in the periphery is enhanced by PD-1 blockade. Based on CD39 and CD103 overexpression, this population may be enriched for tumor-antigen-reactive T cells. While this requires confirmation, this underscores the importance of monitoring this accessible population as an indicator of productive antitumor immunity, and the regulation of this subset, as these circulating granzyme-expressing cells are highly enriched within the proliferative population.

Third, we nominate 2 distinct mechanisms that may regulate cytotoxic CD4^+^ T cells. PD-1 blockade resulted in some peripheral proliferation of these cytotoxic effector cells and an inverse correlation between proliferation and lower levels of KLRG1. However, most cytotoxic cells remained inhibited by KLRG1 after PD-1 blockade, which can act on these cells in several ways. KLRG1 on circulating cytotoxic cells can be directly engaged by E-cadherin on other blood immune subsets such as myeloid dendritic cells, which can contribute to KLRG1-dependent suppression (31). Although KLRG1 levels were lower on T cells in tumors compared with blood, a proportion of T cells maintained KLRG1 expression in tumors (Figure 4D and Figure 7C) and can still be inhibited by tumor E-cadherin. Finally, although bladder cancer can downregulate E-cadherin during epithelial-mesenchymal transition, pathologic series have confirmed that up to half of high-grade/invasive urothelial carcinomas still retain E-cadherin protein expression, and our own flow cytometry confirms retention of E-cadherin in a proportion of tumor cells (Figure 7C), indicating that urothelial carcinoma can still initiate KLRG1-dependent inhibition. Overall, our ability to initiate autologous tumor killing by blocking KLRG1’s interactions with E-cadherin nominate KLRG1 not just as a marker of cytotoxic CD4^+^ T cells with shared tumor specificity, but as an additional regulatory step (separate from PD-1) that can further mobilize killing.

While we believe these findings are novel for cytotoxic CD4^+^ T cells, these are consistent with known functional characteristics of KLRG1-expressing T cell subsets in multiple contexts (32–35). Separately, genetic reporters of KLRG1 expression have identified KLRG1^+^ effector CD8^+^ cells that downregulate KLRG1 and differentiate into longer-lived “exKLRG1” progeny, which may represent the downstream fate of circulating, proliferating cytotoxic CD4^+^ T cells as they lose KLRG1. It seems clear that KLRG1 regulation is context dependent, as in tumors its expression is lower, not confined to cytotoxic CD4^+^, and not correlated with very high PD-1 and Tim-3 expression, which globally marks exhausted intratumoral cells. The dissociation between high immune checkpoint and low KLRG1 expression in tumors suggests that KLRG1 does not mark exhausted T cells, in agreement with other studies (36). Further validation of KLRG1 as a therapeutic target for human circulating cytotoxic CD4^+^ T cells, beyond work in murine models (37, 38) and proliferation of human CD8^+^ T cells in culture (11, 31), would require direct demonstration that intratumoral cytotoxic CD4^+^ T cells are recruited from blood, and that these clonally expanded effector cells in fact recognize tumor antigens. These can be shared antigens, or MHC class II–restricted neoantigens as has been demonstrated for cytotoxic CD4^+^ TILs in patients with bladder cancer (39).

Finally, we clarify the distinction and ontogeny of distinct granzyme-expressing subsets, which has not been studied for cytotoxic CD4^+^ T cells, and poorly studied for CD8^+^ T cells. Our data indicate on one hand that the GZMB-expressing subset is more cytolytic, is more likely to proliferate after PD-1 blockade and home to tumors, and expresses higher levels of KLRG1 and are therefore subject to additional layers of regulation that can be therapeutically reversed. It will be of interest to see which specific subsets of circulating GZMB-expressing CD4^+^ T cells exhibit properties of precursor exhausted T cells that can be mobilized by immunotherapy. Although the overall *GZMB*^+^ population expressed low levels of *TCF7* (Figure 4A), only a small proportion of these cells proliferated after PD-1 blockade, the properties of which will require investigation. However, many questions are raised about their counterparts that only express GZMK; in our data, these are not cytolytic, have higher basal proliferation in patients with cancer, but do not proliferate after immunotherapy, and adopt a memory phenotype in the periphery. At the same time, we see a significant number of GZMK^+^ cytotoxic CD4^+^ (and CD8^+^) T cells with identical antigenic specificity that accumulate in tumors, which our data suggest are derived from the newly activated GZMB^+^CD4^+^ cells. Possible effector functions of this GZMK^+^ subset, and how these may be regulated, require further study. Additional fate mapping in preclinical models would be required to definitively determine the ontogeny, origin, and direction of homing of cytotoxic CD4^+^ T cells.

Interpretation of our study is subject to limitations. The sample size of the scRNA-seq data set is limited, and lacks paired pre-atezolizumab tumors. However, key findings regarding developmental stage, effector and immune checkpoint protein expression, and killing function of cytotoxic CD4^+^ T cells are validated in nonoverlapping patient cohorts distinct from the scRNA-seq data. Cytotoxic CD4^+^ cells may be subject to additional regulatory interactions (beyond the KLRG1/E-cadherin axis) with other suppressive elements of the tumor microenvironment; in addition, N-cadherin, another KLRG1 ligand, becomes upregulated when E-cadherin is downregulated during epithelial-mesenchymal transition, and N-cadherin’s relevance as a functional inhibitory KLRG1 ligand remains to be explored. Nevertheless, blocking E-cadherin alone was sufficient to increase killing of tumor cells by T cells. We chose to block E-cadherin instead of KLRG1, as commercial antibodies against KLRG1 are not well characterized for their functional properties, may activate the receptor, and would require testing and optimization to determine whether KLRG1 blockade yields similar outcomes to blocking E-cadherin. Finally, further examination of longitudinal samples from immunotherapy-treated patients with bladder cancer will identify the long-term evolution of tumor immune recognition in patients who remain disease free, and how this may be subverted in patients who progress after neoadjuvant therapy and cystectomy.

## Methods

### Sex as a biological variable.

Analyses included both male and female patients with bladder cancer. Sex was not explicitly considered as a variable in analyses.

### Study design.

The main objective was to understand the function and regulation of cytotoxic CD4^+^ T cells that circulate between tumor and blood of patients with bladder cancer.

For single-cell analysis of circulating immune responses, the sample set was matched blood from 6 of 7 patients with localized, muscle-invasive bladder cancer whose tumor and NAT scRNA-seq data were previously published (7). These included 3 patients (“Anti-PD-L1 B,” “Anti-PD-L1 C,” and “Anti-PD-L1 D”) treated with neoadjuvant atezolizumab prior to cystectomy on a trial (see *Clinical trial design* below), 1 patient treated with neoadjuvant chemotherapy before cystectomy as standard of care, and 2 patients untreated with any systemic therapy before cystectomy. Blood samples from an additional patient from the neoadjuvant trial (“Anti-PD-L1 E”), as well as 3 separately sequenced technical replicates from a single healthy donor, were also analyzed but without matching tumor data. For standard of care, there was a single blood draw the same day as surgery. For trial patients, a baseline blood draw was taken prior to starting (pretreatment) and after neoadjuvant atezolizumab (posttreatment) for the trial. All posttreatment blood draws except 1 were prior to surgery, and most were within 1–2 weeks of surgery. scRNA-seq and scTCR-seq were conducted as described below, and jointly analyzed with the published tumor data. All available blood time points and a single tumor time point (surgery) were combined for clustering and initial analysis.

For flow cytometry, standard-of-care patients with localized muscle-invasive bladder cancer who had not previously received systemic therapy (*n* = 8 blood samples and 6 tumor samples distinct from our scRNA-seq data, Figures 4 and 5) were collected on an IRB-approved biobanking protocol for staining to determine their functional status. Flow cytometric analysis of paired pre- and postimmunotherapy blood samples from our neoadjuvant clinical trial (Figure 6 and Supplemental Figure 6) were conducted on 14 trial participants who had available samples (10 trial patients were distinct from those in our scRNA-seq data), along with 8 available healthy donor blood samples. For these flow cytometric analyses, no prespecified statistical hypotheses were applied during selection of these samples.

For functional experiments (Figure 7 and Supplemental Figure 7), data are presented from 3 distinct standard-of-care patients with localized, muscle-invasive bladder cancer who had not previously received systemic therapy, whose CD4^+^ T cells from blood and/or tumor were successfully expanded in culture with IL-2, -7, -15, and -21, and had additional paired aliquots of viably cryopreserved tumor cells as targets for killing assays.

### Clinical trial design.

Neoadjuvant clinical trial NCT02451423 is a completed single-arm, open-label, phase II trial at UCSF where patients received 1, 2, or 3 doses of atezolizumab (anti–PD-L1) every 3 weeks prior to cystectomy. After planned dose escalation where 6 patients were treated sequentially at each dose level and followed for safety, additional patients were treated in dose expansion with 3 preoperative doses. Clinical response was determined as pathologic downstaging to less than ypT2 (i.e., ypT0/Ta/T1), and absence of node-positive disease, in the cystectomy specimen after neoadjuvant therapy. All patients were followed for up to 2 years after surgery for safety and clinical outcomes until progressive disease, death, or withdrawal of consent. Key inclusion criteria were patients with localized, resectable urothelial carcinoma (T2-T4a, N0-1, M0) who were unable to receive cisplatin-based treatment (estimated glomerular filtration rate <60 mL/min, grade ≥2 neuropathy/hearing loss, patient decision). Clinical demographics are presented in Supplemental Dataset 1.

### Blood processing.

Whole blood was collected in heparinized tubes and processed within 24 hours. Whole blood was diluted 1:1 with PBS (Ca^2+^ and Mg^2+^ free) and centrifuged on a Ficoll gradient. PBMCs were removed from the white cell interface and washed twice with PBS before freezing in 10% DMSO and 12.5% human serum albumin in RPMI-1640.

### Tumor processing.

Cystectomy specimens were obtained fresh from the operating field, and dissected in pathology where grossly apparent tumors or adjacent bladder not grossly affected by tumors (“normal adjacent tissue”) were isolated, minced, and transported at room temperature in L15 media with 15 mM HEPES and 600 g/L glucose. These were further minced and digested using freshly prepared 200 mg/mL Liberase (Roche) and 100 mg/mL DNase (Roche) in RPMI-1640 at 37°C for 1 hour with a gentleMACS dissociator (Miltenyi Biotec). Cells were washed with 10% heat-inactivated FBS in RPMI-1640 and passed through a 100-μm cell strainer. Single-cell suspensions were counted for viability and stained for cell sorting.

### scRNA-seq.

CD3^+^CD4^+^ and CD3^+^CD8^+^ T cells were sorted from digested tumors and nonmalignant tissues, or Ficoll purified and previously cryopreserved PBMCs from the same patients, into 500 mL of PBS with 0.04% BSA. Double-positive (CD4^+^ and CD8^+^ by protein expression) cells were excluded during sorting and did not contribute to observed cytotoxic CD4^+^ (single-positive) T cells. Cells were centrifuged and resuspended at 1000 cells/mL, and all sorted cells were used without exceeding the maximum number recommended by the manufacturer. Droplet-based scRNA-seq was performed using 10× Genomics Chromium Single Cell 3′ (v1), with sequencing on an Illumina HiSeq 2500 (Rapid Run).

### scTCR-seq.

For our overall scTCR-seq strategy, targeted PCR with NEBNext Ultra II Q5 Master Mix (New England Biolabs) used a pool of forward Vα and Vβ primers containing the TruSeq Read 1 primer sequence with a reverse P7 primer to amplify CDR3 sequences as well as the 10× Genomics barcodes. A second PCR with forward primers containing the Illumina P5, i5, and TruSeq Read 1 sequences was used with reverse P7 primer to generate TCR libraries for sequencing. Sequencing used an Illumina NovaSeq S1 with separate lanes for TCRα and TCRβ sequencing. Read 1 contained 280 bp of the TCRa and TCRb CDR3 sequence, and the i7 read contained the 14 bp 10× Genomics barcode.

For specific results validating enrichment of tumor-matched TCRαβ clones in sorted KLRG1^+^CD4^+^ T cells from PBMCs (patients 50 and 65, Supplemental Dataset 10), 10× Genomics Human Single Cell V(D)J reagents were used.

### scRNA-seq analysis.

10× Genomics sequencing data were processed through the Cell Ranger pipeline (v1.1, hg19 genome assembly) with default settings and filtered gene-barcode matrices were analyzed using scanpy (40). Gene expression values were transformed (log_2_ + 1) and normalized to 10,000 counts per cell. Cells with mitochondrial gene expression of greater than 10% were filtered out. The resulting matrix was batch corrected by regressing out total UMI counts and percentage mitochondrial genes using the scanpy function followed by scanpy implementation of ComBat (41). The adjusted matrix was scaled to a mean of zero and variance of 1. Highly variable genes were selected using scanpy followed by principal component analysis (PCA), Leiden clustering, and UMAP plotting with default settings. CD4^+^ and CD8^+^ T cells were clustered together at a resolution of 0.9. DE analysis was carried out to identify marker genes that were upregulated in each individual cluster relative to all other single cells. Annotation of each unbiased population was performed by manual DE gene inspection.

### scTCR-seq analysis.

For our overall scTCR-seq strategy, 10× Genomics TCR barcodes were matched with 10× Genomics barcodes from cells with RNA sequences that passed filtering in the Cell Ranger pipeline. TRA and TRB CDR3s reads were aligned against known TRA and TRB CDR3 sequences and assembled into clonotype families using miXCR (42). For any given 10× Genomics barcode, the most abundant TRA or TRB clonotype was accepted for further analysis; if 2 TRA or TRB clonotypes were equally abundant for a given 10× Genomics barcode, the clonotype with highest sequence alignment score was used for further analysis. Cells with paired TRA and TRB were used for downstream analysis.

For specific results validating enrichment of tumor-matched TCRαβ clones in sorted KLRG1^+^CD4^+^ T cells from PBMCs (patients 50 and 65, Supplemental Dataset 10), 10× Genomics Cell Ranger pipeline v8.0.1 was used.

### TCR network analysis.

Network analysis was done at the cellular level. Cells without phenotypic information from scRNA-seq were excluded from analysis. First, for each patient (including all samples from different specimens and different time points) in each chain (α and β), the Levenshtein distance (R package RecordLinkage; ref. 43) was calculated pairwise (each pair of cells) to obtain the distance matrix by using Spark (R package sparklyr, https://spark.rstudio.com) for parallel computing. Next, the 2 distance matrices of α and β chains were combined while subgrouping into different components (CD4^+^ and CD8^+^ cells) and different specimen types (blood and tumors). We then identified cells as either matched or nonmatched (between blood and tumor) based on the combined matrix for each patient; or either expansion (degree > 0) versus nonexpansion (degree = 0). The network was built based on patient level by component type (CD4^+^ and CD8^+^ cells) and by specimen type (blood and tumor); only TCRs with the same amino acids in both α chains and β chains were connected (distance = 0). Network analysis was plotted by using R packages igraph (44) and ggraph. The number of clusters belonging to each network and the maximum cluster size (the maximized number of clones belonging to each cluster across all clusters within the network) were used to describe the network properties. Both values were normalized by the number of cells and log_10_ transformed. This analysis package is available at https://github.com/mlizhangx/Network-Analysis-for-Repertoire-Sequencing

### Pseudotime analysis.

Pseudotime analysis was performed using Monocle v2.10.1 (45).

### Flow cytometry.

For generating RNA-seq libraries, freshly dissociated TILs and previously cryopreserved pre- or postimmunotherapy PBMCs were stained for 30 minutes on ice in the dark with the following anti-human antibodies: Brilliant Ultraviolet 395–CD45 (Becton Dickinson, clone H130); and from BioLegend, Brilliant Violet 421–CD4 (clone OKT4), Brilliant Violet 650–CD3 (clone UCHT1), Alexa Fluor 647–CD8 (clone SK1), and Draq7 (cell viability dye). Cells were washed twice and resuspended in SORT buffer (2% heat-inactivated FBS, 1 mM EDTA, 25 mM HEPES at pH 7, and 100 U/mL penicillin and streptomycin in PBS). Cells were filtered through a cell strainer before sorting for Draq7^–^CD45^+^CD3^+^CD4^+^ or Draq7^–^CD45^+^CD3^+^CD8^+^ cells on a FACSAria Fusion (Becton Dickinson).

For flow validation, previously cryopreserved samples were thawed, stained with Live/Dead Fixable Near-IR stain (Invitrogen, L34975), washed with FACS buffer (2% heat-inactivated FBS and 1 mM EDTA in PBS), and surface stained for 30 minutes on ice in the dark with the following: from Becton Dickinson, Brilliant Violet 480–CD3 (clone UCHT1), Brilliant Ultraviolet 805–CD45 (clone HI30), Brilliant Ultraviolet 395–CD4 (clone RPA-T4), Brilliant Ultraviolet 496–CD8 (clone RPA-T8), APC-R700–CCR7 (clone 3D12), and Brilliant Ultraviolet 737–PD-1 (clone EH12.1); and from BioLegend, Brilliant Violet 650–CD45RA (clone HI100), Brilliant Violet 785–CD25 (clone BC96), and Brilliant Violet 421–KLRG1 (clone SA231A2). After washing, cells were fixed and permeabilized using the eBioscience FoxP3/Transcription Factor Staining Buffer and intracellular staining was carried out for 30 minutes at room temperature in the dark with following: from Becton Dickinson, Brilliant Violet 510–GZMB (clone GB11) and PE-CF594–FoxP3 (clone 259D/C7); and from BioLegend, FITC-GZMK (clone GM26E7) and Brilliant Violet 605–Ki67 (clone Ki-67). After staining, cells were washed and fixed with FluoroFix buffer (BioLegend). Cells were acquired the next day on a FACSymphony (Becton Dickinson) using FACSDiva software with single-channel compensation controls acquired on the same day. Data were analyzed using FlowJo analysis software (Becton Dickinson).

For sorting T and tumor cells for T cell expansion and killing, cryopreserved PBMCs or tumor was thawed, washed, and stained with Near-IR dead cell stain (Invitrogen, L34975), and washed and stained with the following antibodies for 30 minutes on ice in the dark: Brilliant Ultraviolet 395–CD45 (Becton Dickinson, clone HI30); and from BioLegend, Brilliant Violet 785–CD3 (clone UCHT1), Brilliant Violet 650–CD8 (clone SK1), Alexa Fluor 488–CD4 (clone OKT4), PE-CD127 (clone A019D5), PE-Cy7–CD25 (clone BC96), and Brilliant Violet 421–KLRG1 (clone SA231A2). Cells were washed twice with SORT buffer, filtered through a cell strainer, and sorted with a FACSAria Fusion (Becton Dickinson). A portion of tumor cells was additionally stained for E-cadherin (Alexa Fluor 647–E-cadherin, BioLegend, clone 67A4) and was not used for sorting.

### T cell expansion and autologous tumor killing assay.

For T cell expansion, CD4^+^ (live CD45^+^CD3^+^CD4^+^[CD127^lo^CD25^+^]^–^) or CD8^+^ (live CD45^+^CD3^+^CD8^+^) KLRG1^+^ and KLRG1^–^ T cells were sorted with a FACSAria Fusion (Becton Dickinson). T cells were activated with Dynabeads human T-activator CD3/CD28/CD137 (Gibco) at 1:5 beads to T cell ratio and cultured in ImmunoCult-XF T cell expansion medium (Stemcell Technologies) with 10% human AB serum (heat inactivated), 100 U/mL penicillin and streptomycin, 50 mg/mL gentamicin, 200 U/mL IL-2 (Peprotech), and 10 ng/mL each of IL-7, -15, and -21 (Peprotech) in a well of a U-bottom 96-well plate and incubated at 37°C, 5% CO_2_ and 95% relative humidity. Half of the media was changed weekly and cells were expanded into 2 wells only when media were orange yellow. With each expansion, IL-2 concentration was increased from 200 to 500 U/mL, and then to 1000–2000 U/mL as needed until sufficient T cells were obtained.

Autologous tumor cells were sorted (live CD45^–^CD3^–^) from another aliquot of frozen tumor cells from the same patient and 3000–5000 cells per well were plated in complete media (RPMI-1640, 1% nonessential amino acids, 1 mM sodium pyruvate, 2 mM L-glutamine, 100 U/mL penicillin and streptomycin, 10% heat-inactivated FBS) in flat-bottom 96-well plate and incubated at 37°C until the next day. Where mentioned, tumor cells were stained first with 3 mM Cytolight Rapid Red (Essen Bioscience, Sartorius) and washed once before plating. Dead T cells were removed from expanded T cells using an EasySep Dead Cell Removal (Annexin V) Kit (Stemcell Technologies) before plating live T cells with tumor cells at a 40:1 ratio and Annexin V Green reagent (Essen Bioscience, 1 mL/200 mL of media) was added. Anti-CD324 (anti–E-cadherin) functional grade (eBioscience, clone DECMA-1) was added to tumor cells (10 mg/mL) and incubated for 30 minutes at room temperature before T cells were added. Where mentioned, rat IgG1, κ isotype control (eBioscence) was used and anti–human HLA-DR, -DP, -DQ (MHC II, clone Tu39, Becton Dickinson) antibody was added 15 minutes before addition of anti–E-cadherin antibody. Cells were incubated at 37°C, 5% CO_2_ and 95% relative humidity, and monitored by IncuCyte Zoom (Essen Bioscience, Sartorius) at 1-hour intervals. Analysis was performed using IncuCyte Zoom software. All traces are displayed as relative change in cell death from time point 0 with background death of T cells at each time point subtracted.

### Statistics.

The following tests were carried out with R software (https://www.r-project.org/): (i) Pairwise comparison of signature gene scores between each phenotypic population. (ii) Pairwise comparison of percentage of cells between each phenotypic population. (iii) Comparison of number of clusters or cluster size between blood and tumor or between matched and nonmatched TCR clusters, by linear mixed modeling for each cell type within CD4^+^ and CD8^+^ cells separately, where log_10_ of number of clusters was considered as an outcome, while specimen type (blood or tumor) and patient were treated as fixed and random effects, respectively. Coefficients in the output represent the direction and magnitude of difference between blood and tumor. Statistical significance was declared at a *P* value of less than 0.05. (iv) DE gene testing was done with MAST (46). The zero-inflated model was applied to cell level while adjusting for covariates, including time points, specimen types, matched versus nonmatched, and number of genes per cell. Multiple testing adjustment was done by controlling false discovery rate. For all box-and-whisker plots, the box extends from the 25^th^ to 75^th^ percentiles. The whiskers extend to the minimum and maximum data points, and the line within the box is the median.

The following statistical tests were carried out with GraphPad Prism 9 for flow cytometry: (i) Comparison of 2 paired cell subsets using Wilcoxon’s matched-pairs signed-rank test. (ii) Comparison of 2 nonpaired cell subsets using the Mann-Whitney *U* test. (iii) Comparison of 3 or more paired cell subsets using Friedman’s test with Dunn’s multiple-comparison test. (iv) Comparison of 3 or more nonpaired cell subsets using the Kruskal-Wallis test with Dunn’s multiple-comparison test.

### Study approval.

All studies were conducted under supervision of the UCSF IRB. Standard-of-care patients were recruited on a biobanking protocol (IRB no. 10-04057); inclusion criteria were patients with localized, muscle-invasive bladder cancer already scheduled to undergo transurethral resection of bladder tumor or surgical cystectomy, and able to consent to research collection. The neoadjuvant immunotherapy clinical trial was conducted under IRB no. 14-15423.

### Data availability.

Processed scRNA-seq and scTCR-seq data have been deposited in the NCBI Gene Expression Omnibus (GEO GSE293860). Code including Jupyter notebooks for preprocessing, processing, and downstream analysis is available at https://github.com/fonglab/JCI-Insight-Kwek-2025 Values for each figure are included with this manuscript as a supplemental Supporting Data Values file.

## Author contributions

SSK, LF, and DYO conceived and designed the study. SP, MVM, and DYO acquired patient samples and clinical data. SSK, AI, EDC, HC, DL, AL, MC, YS, and DYO carried out experiments and data acquisition. SSK, HY, TL, EDC, LZ, EM, CJY, LF, and DYO performed data analysis and interpretation. SSK, AS, LF, and DYO wrote and revised the manuscript.

## Supplementary Material

Supplemental data

Supplemental data set 1

Supplemental data set 2

Supplemental data set 3

Supplemental data set 4

Supplemental data set 5

Supplemental data set 6

Supplemental data set 7

Supplemental data set 8

Supplemental data set 9

Supplemental data set 10

Supporting data values

## Version 1 04/29/2025

In-Press Preview

## Version 2 06/09/2025

Electronic publication
