# Supplementary material for: Identification and regulation of circulating tumor-TCR-matched cytotoxic CD4+ lymphocytes by KLRG1 in bladder cancer
Source: JCI Insight. 2025 Apr 29;10(11):e177373. doi: 10.1172/jci.insight.177373 (PMC12220972; doi:10.1172/jci.insight.177373)
Supplement: Supplemental data [file jciinsight-10-177373-s093.pdf]

Supplemental Figures:

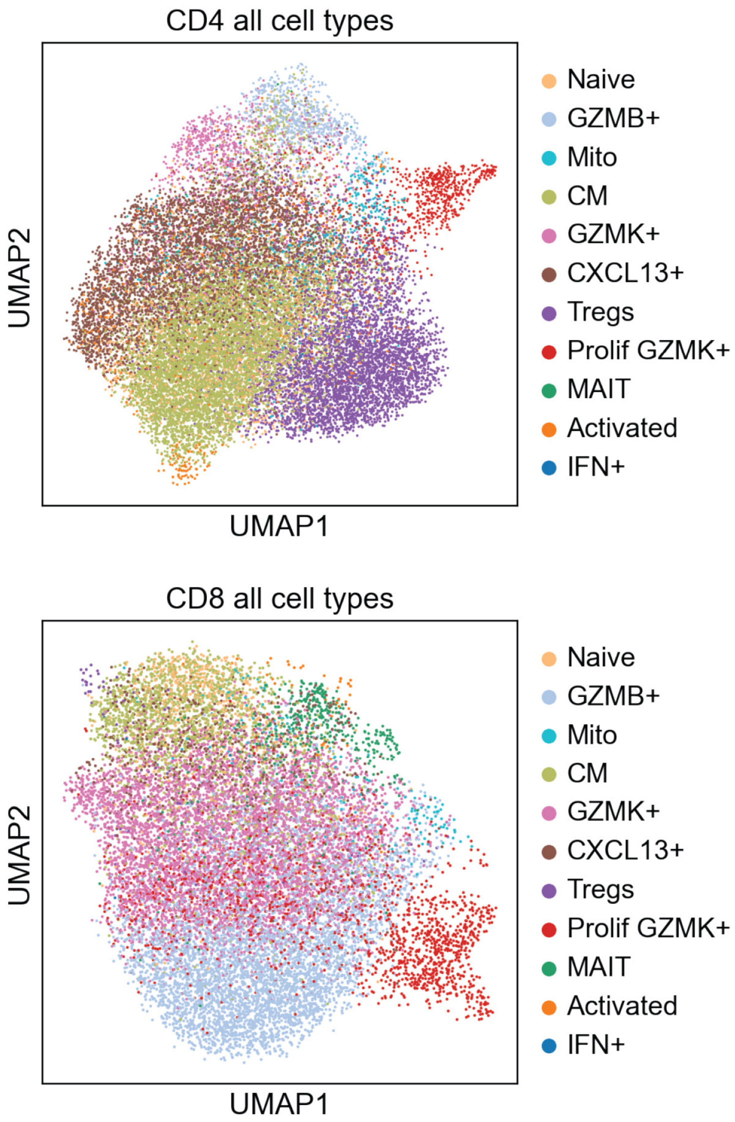

Supplemental Figure 1. Reclustering of CD4⁺ and CD8⁺ as distinct populations confirms the distinct identity of cytotoxic CD4⁺ populations. Identical workflows were applied as in **Figure 1B**, except that the input cells for this clustering were either CD4⁺ or CD8⁺ alone, and the results of this separate clustering are plotted in new UMAP space**.** Prior annotations from jointly clustering (**Figure 1B**) were transferred to these cells and are shown in color.

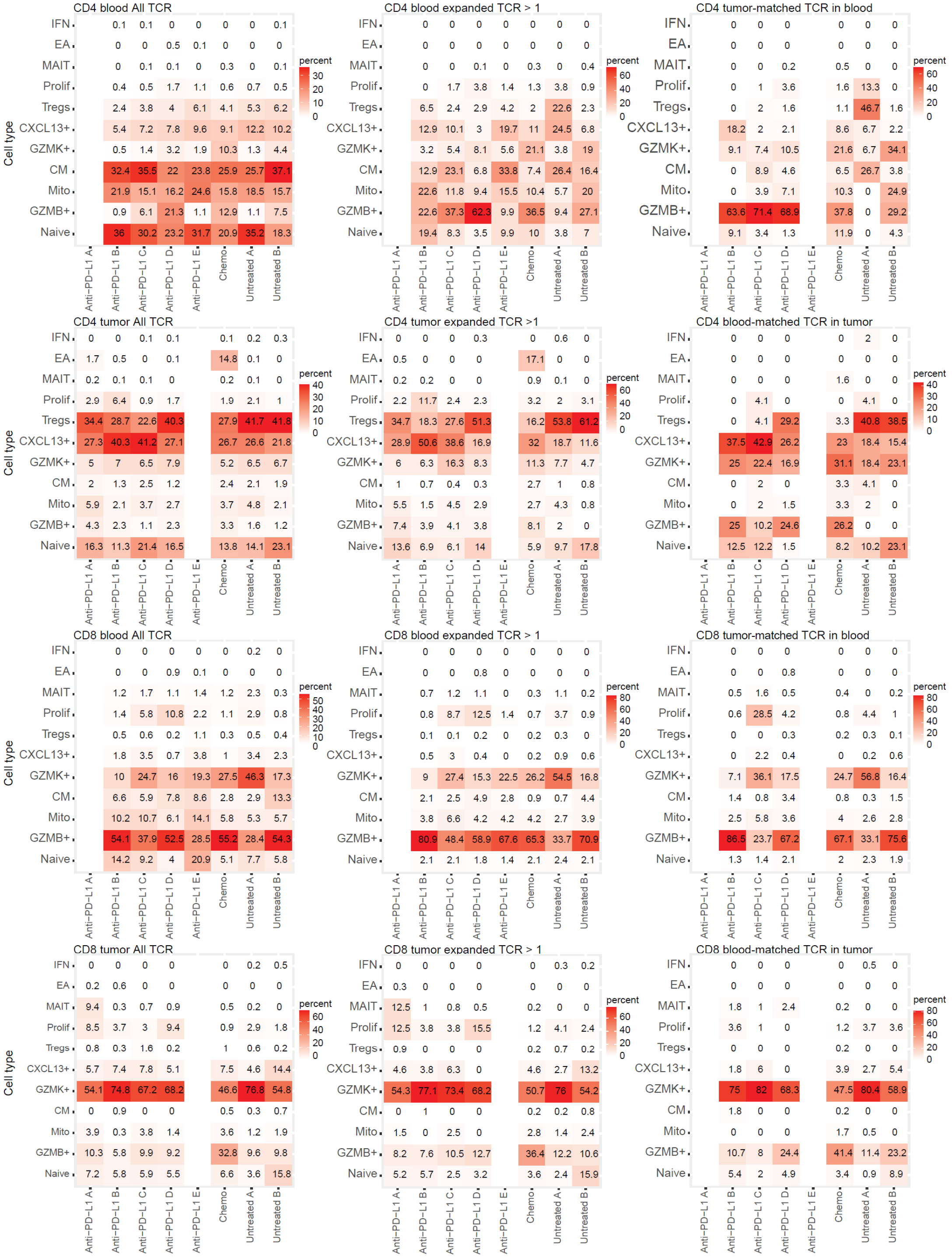

Supplemental Figure 2. Percentages of each phenotypic population of cells with all TCR, expanded TCR, and blood-tumor matched TCR in CD4 and CD8 for each patient. Each heatmap plots the normalized percentage of cell counts in each cell type (row) for each patient (column).

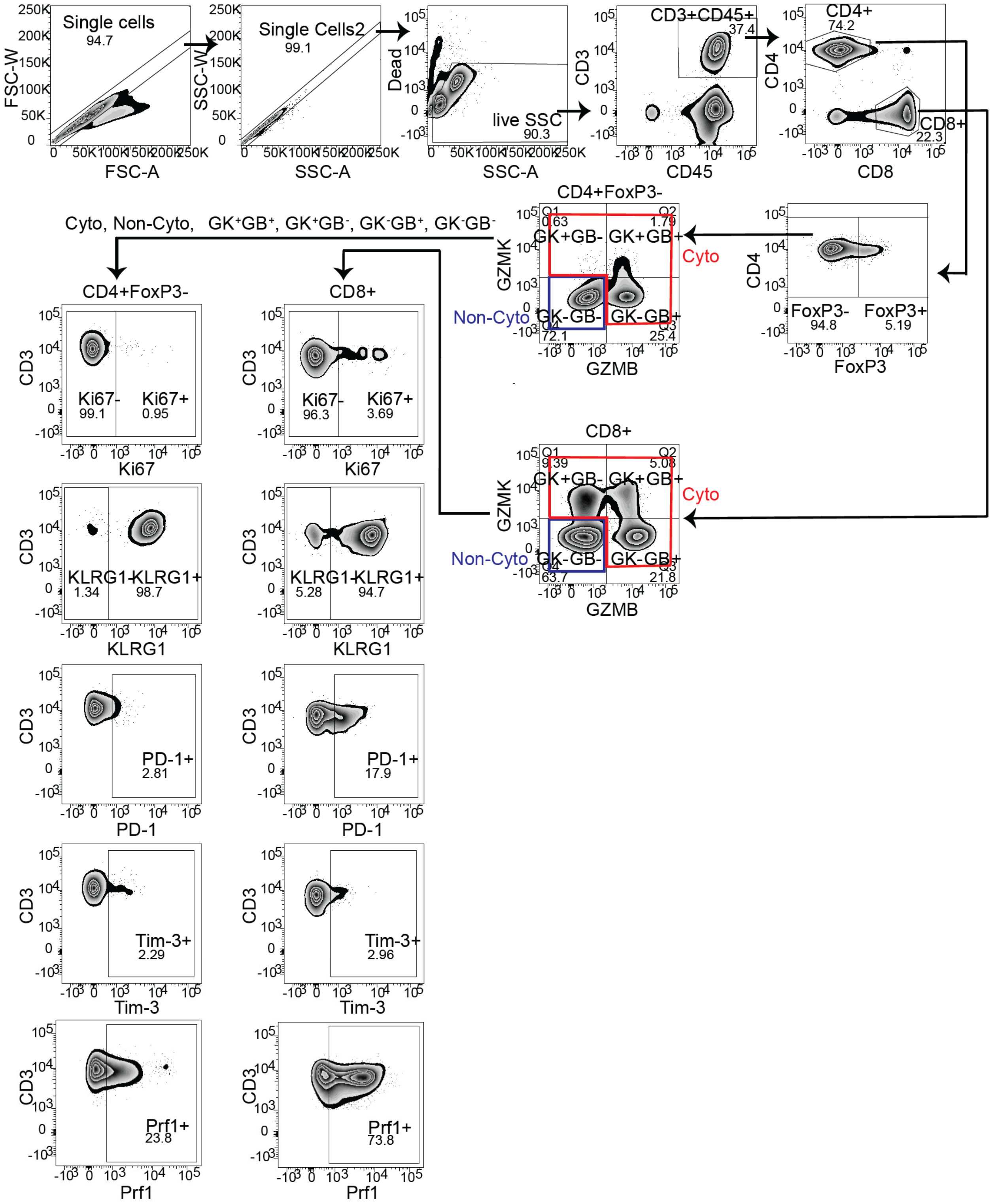

Supplemental Figure 3. **Gating strategy & representative flow plots for Figures 4 and 5 and Supplemental Figure 4.**

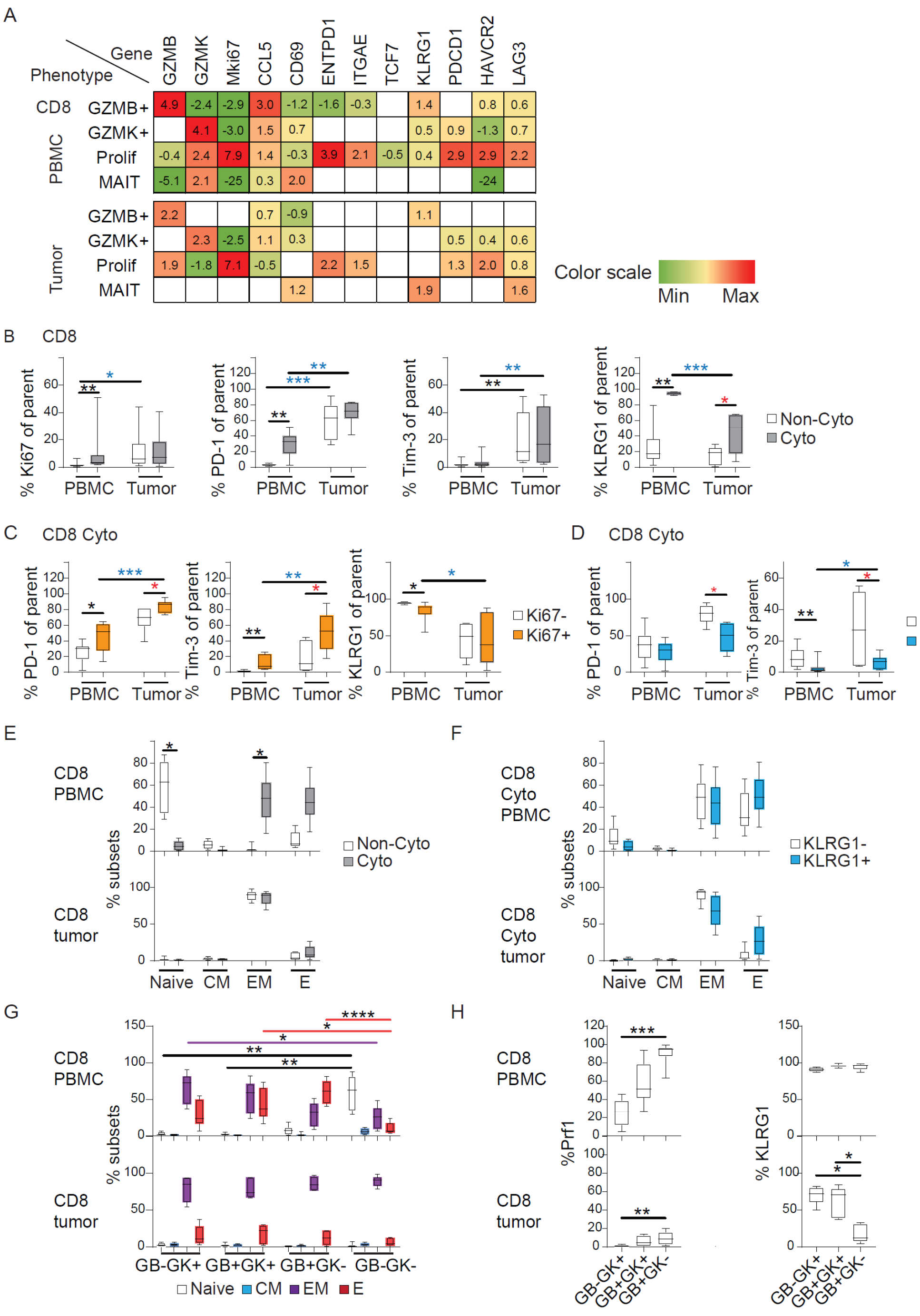

Supplemental Figure 4. Expression of proliferative, inhibitory markers and perforin on different cytotoxic subsets and developmental stages in CD8$^+$ from PBMC and bladder tumor. (**A**) Normalized log fold change from scRNAseq analysis for selected phenotypes and genes. Graphs were obtained by flow cytometry of 8 PBMC and 6 tumors from SOC bladder cancer patients as for CD4$^+$ T cells in Figures 4 and 5. All graphs in this figure are box plots with min and max for CD8$^+$ T cells from PBMC and tumor showing (**B**) % Ki67$^+$, PD-1$^+$, Tim-3$^+$, and KLRG1$^+$ of total cytotoxic (Cyto) and non-cytotoxic subtypes (Non-Cyto); (**C**) % PD-1$^+$, Tim-3$^+$, and KLRG1$^+$ of Ki67$^+$ and Ki67$^-$ of cytotoxic cells; (**D**) % PD-1$^+$ and Tim-3$^+$ of KLRG1$^+$ and KLRG1$^-$ of cytotoxic cells; (**E**) proportion of developmental subsets in Cyto and Non-cyto cells; (**F**) proportion of developmental subsets in KLRG1$^-$ and KLRG1$^+$ cytotoxic cells; (**G**) proportion of developmental subsets in each cytotoxic subtypes (red, purple and black lines indicate significant pairwise comparison of proportion of E,

EM or Naïve subsets respectively between cytotoxic subtypes); and (**H**) % $Prf1^+$ and $KLRG1^+$ of each cytotoxic subtype. Comparison of paired cell subsets within PBMC or tumor was performed using Friedman test with Dunn's multiple comparisons. Comparison of non-paired cell subsets between PBMC and tumor was performed using Kruskal-Wallis test with Dunn's multiple comparisons. For (B) to (D), asterisks in black, red or blue indicate significant differences between subsets within PBMC, within tumor, and between PBMC and tumor respectively.

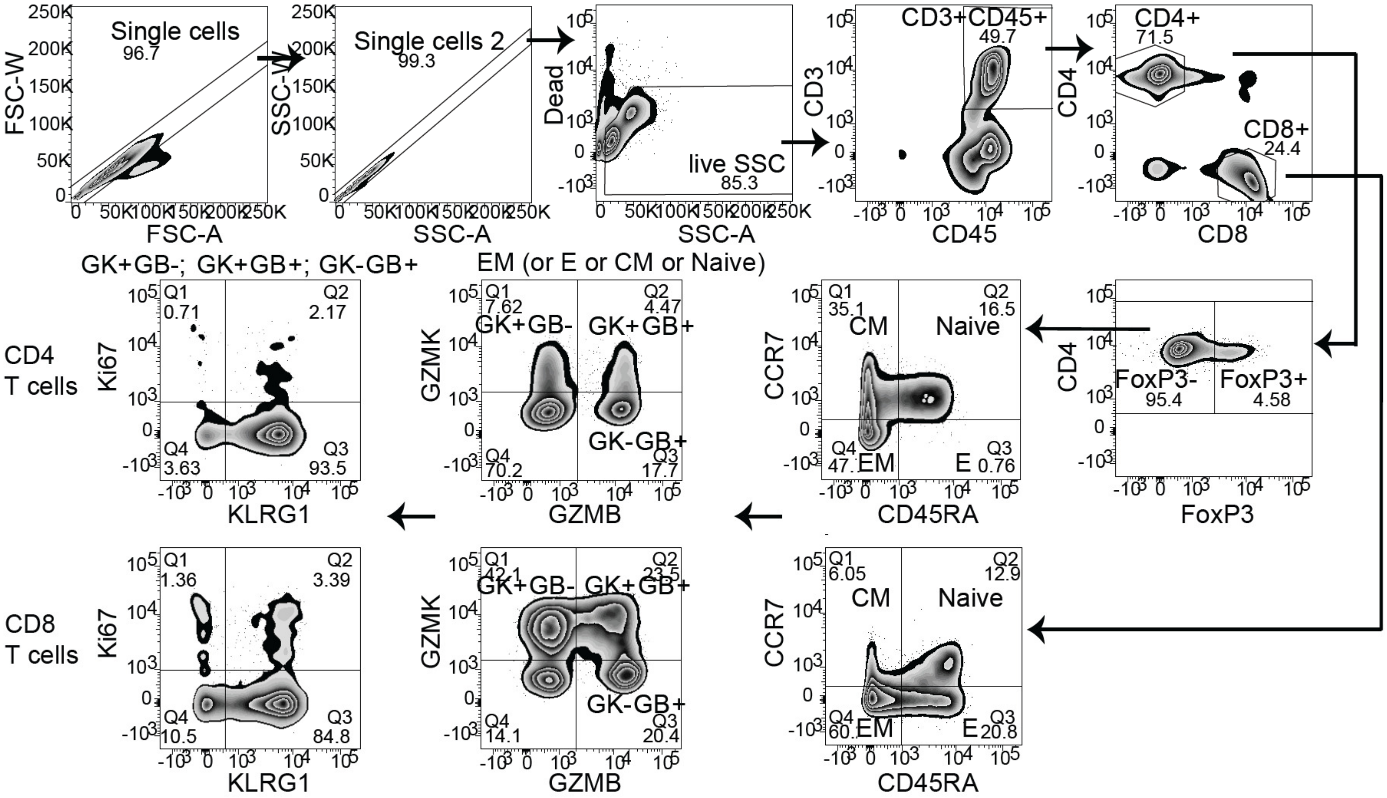

Supplemental Figure 5. Gating strategy & representative flow plots for Figure 6 and Supplemental Figure 6.

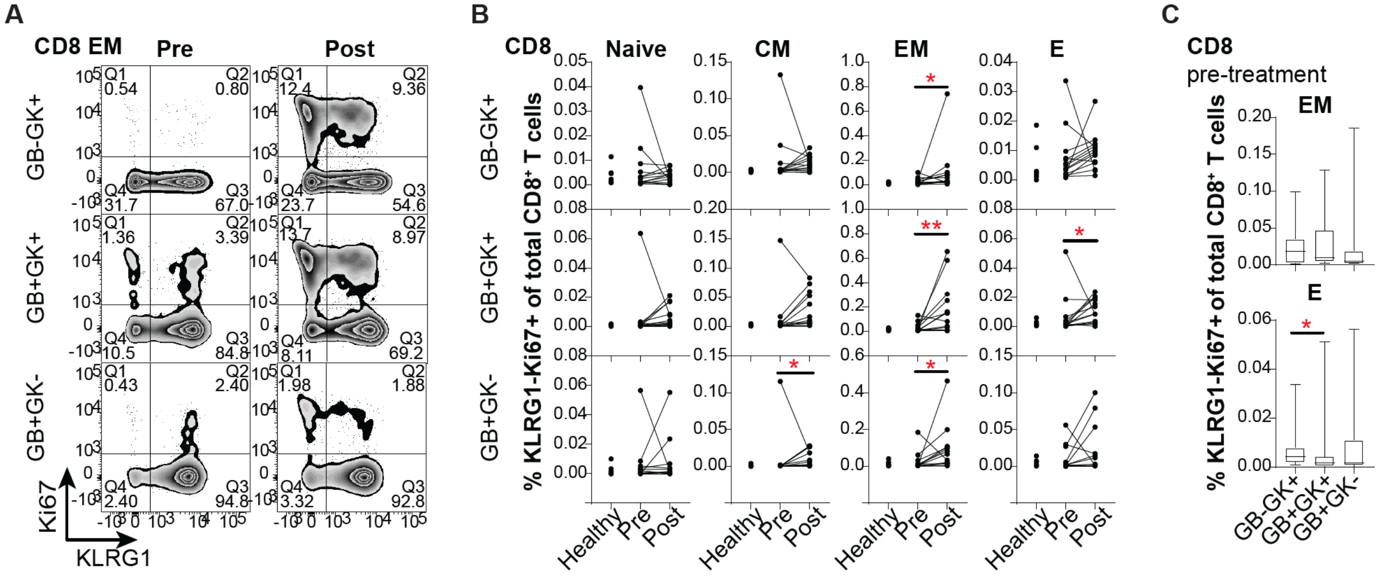

Supplemental Figure 6. Anti-PD-L1 treatment increased Ki67 and decreased KLRG1 expression on cytotoxic CD8$^+$ T cells in the blood. Flow cytometry was carried out on PBMC from 8 healthy individuals and 14 pre- and post-Atezo treated bladder cander patients (clinical trial NCT02451423). (**A**) Representative flow cytometry plots of pre and post-treatment CD8$^+$ EM cytotoxic T cell subsets showing Ki67 and KLRG1 expression. (**B**) Plots showing % KLRG1$^-$Ki67$^+$ of total CD8$^+$ T cells in cytotoxic subtypes with naïve-like, CM, EM and E phenotypes. (**C)** Comparisons of pre-treatment % KLRG1$^-$Ki67$^+$ of total CD8$^+$ T cells between cytotoxic subtypes. Mann-Whitney U test was carried out for comparison between healthy and pre-treatment cancer samples; Wilcoxon matched-pairs signed rank t test was carried out for comparison between pre and post-treatment cancer samples.

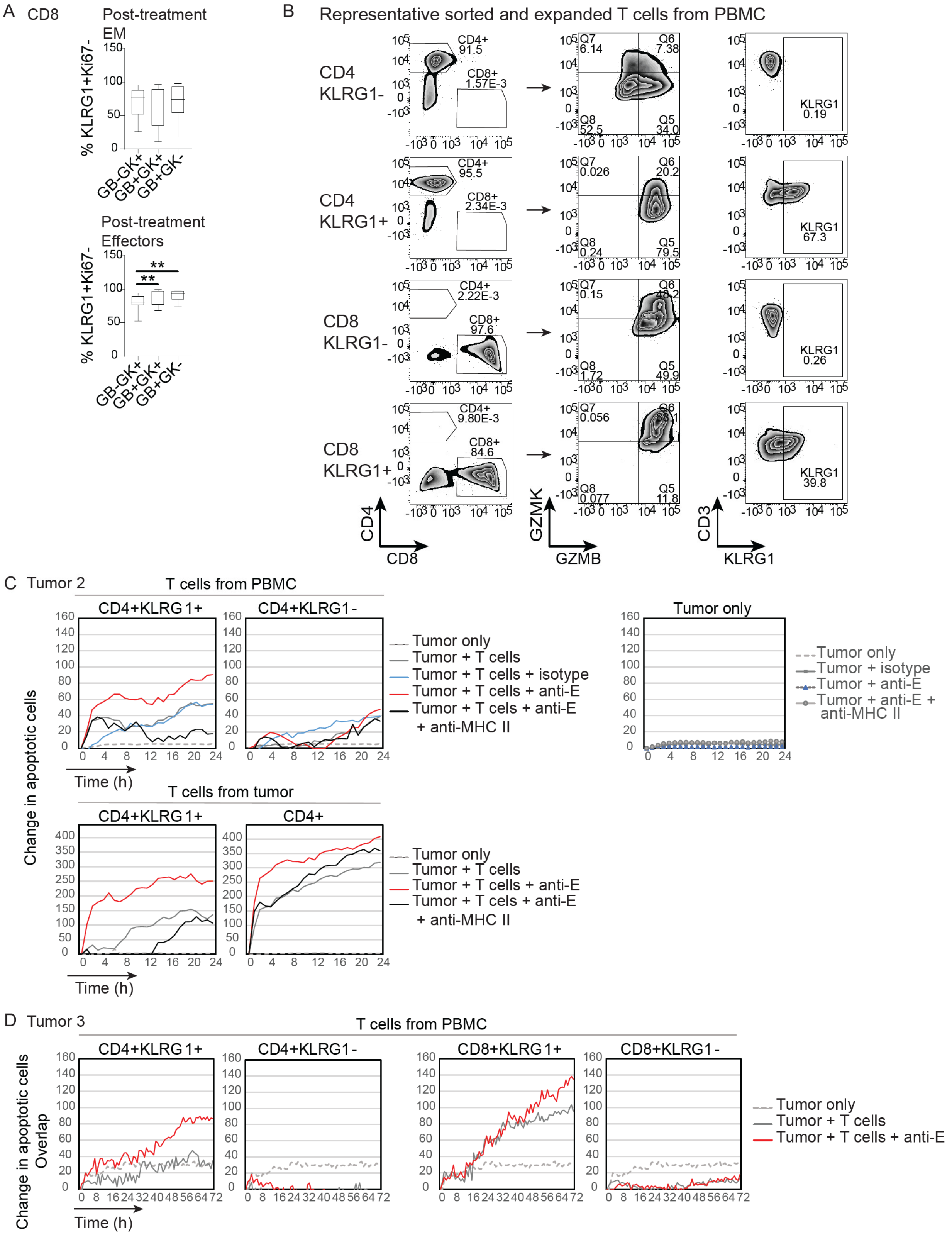

Supplemental Figure 7. Autologous tumor killing experiment by T cells for tumor 2 and 3. (**A**) Box plot with min and max showing % KLRG1^{+}Ki67^{-} of 14 post-Atezo treatment CD8^{+} EM and effectors cytotoxic subsets in PBMC. (**B**) Representative flow plots of expanded cells from sorted KLRG1 positive and KLRG1 negative cells from CD4^{+} and CD8^{+} T cells. (**C**) Apoptotic tumor cell death plotted as the relative change in annexin A green fluorescent positive cell count from time point zero with background death of T cells at each time point subtracted for tumor 2. (**D**) Apoptotic tumor cell death plotted as the relative change in overlap

signals of annexin A green fluorescent and Cytolight Rapid Red positive cell count from time point zero for tumor 3.
